# Health system utilization before age 1 among children later diagnosed with autism or ADHD

**DOI:** 10.1038/s41598-020-74458-2

**Published:** 2020-10-19

**Authors:** Matthew M. Engelhard, Samuel I. Berchuck, Jyotsna Garg, Ricardo Henao, Andrew Olson, Shelley Rusincovitch, Geraldine Dawson, Scott H. Kollins

**Affiliations:** 1 Department of Psychiatry and Behavioral Sciences, Duke University School of Medicine, 2608 Erwin Rd, Durham, NC 27705 USA; 2 Department of Statistical Science, Duke University, Durham, NC USA; 3 Duke Forge, Duke University School of Medicine, Durham, NC USA; 4 Duke Clinical Research Institute, Duke University School of Medicine, Durham, NC USA; 5 Department of Biostatistics and Bioinformatics, Duke University School of Medicine, Durham, NC USA; 6 Duke Center for Autism and Brain Development and Duke Institute for Brain Sciences, Durham, NC USA

**Keywords:** ADHD, Autism spectrum disorders, Health services, Risk factors

## Abstract

Children with autism spectrum disorder (ASD) or attention deficit hyperactivity disorder (ADHD) have 2–3 times increased healthcare utilization and annual costs once diagnosed, but little is known about their utilization patterns early in life. Quantifying their early health system utilization could uncover condition-specific health trajectories to facilitate earlier detection and intervention. Patients born 10/1/2006–10/1/2016 with ≥ 2 well-child visits within the Duke University Health System before age 1 were grouped as ASD, ADHD, ASD + ADHD, or No Diagnosis using retrospective billing codes. An additional comparison group was defined by later upper respiratory infection diagnosis. Adjusted odds ratios (AOR) for hospital admissions, procedures, emergency department (ED) visits, and outpatient clinic encounters before age 1 were compared between groups via logistic regression models. Length of hospital encounters were compared between groups via Mann–Whitney *U* test. In total, 29,929 patients met study criteria (ASD N = 343; ADHD N = 1175; ASD + ADHD N = 140). ASD was associated with increased procedures (AOR = 1.5, *p* < 0.001), including intubation and ventilation (AOR = 2.4, *p* < 0.001); and outpatient specialty care, including physical therapy (AOR = 3.5, *p* < 0.001) and ophthalmology (AOR = 3.1, *p* < 0.001). ADHD was associated with increased procedures (AOR = 1.41, *p* < 0.001), including blood transfusion (AOR = 4.7, *p* < 0.001); hospital admission (AOR = 1.60, *p* < 0.001); and ED visits (AOR = 1.58, *p* < 0.001). Median length of stay was increased after birth in ASD (+ 6.5 h, *p* < 0.001) and ADHD (+ 3.8 h, *p* < 0.001), and after non-birth admission in ADHD (+ 1.1 d, *p* < 0.001) and ASD + ADHD (+ 2.4 d, *p* = 0.003). Each condition was associated with increased health system utilization and distinctive patterns of utilization before age 1. Recognizing these patterns may contribute to earlier detection and intervention.

## Introduction

Autism spectrum disorder (ASD) and attention deficit hyperactivity disorder (ADHD) affect approximately 1.5%^1^ and 11%^2^ of children in the United States, respectively, and ADHD symptoms are present in 40–60% of children with ASD^3,4^. Both diagnoses lead to higher rates of healthcare utilization^5–9^ and place considerable financial burden on affected individuals and their families^10–17^. Rates of hospitalization and outpatient clinic visits among children with ASD are approximately twice that of the average child, contributing to a two- to threefold increase in annual costs^18^ comparable to other chronic medical diagnoses^13^. Children with ADHD visit the emergency department (ED) approximately twice as often as those without^7^, with even higher rates reported among those with comorbid psychiatric conditions^19^. These factors are compounded in children with co-occurring ASD and ADHD, who incur higher healthcare costs than children with either disorder alone^20^.

These findings demonstrate that individuals with ASD and ADHD utilize greater healthcare resources once diagnosed, but little is known about their health system utilization early in life, prior to diagnosis. Emerging evidence suggests that diagnosis is preceded by distinct patterns of increased utilization. For example, a study of newly diagnosed ADHD patients in Germany found that healthcare costs were elevated in the year before diagnosis^21^. More recently, analysis of nationwide registry data from Denmark demonstrated that rates of medical service use were 1.8 times higher in children later diagnosed with ADHD, and co-occurring ASD further increased this difference^22^. These results suggest that increased utilization may be characteristic of ASD and/or ADHD, and not simply a consequence of recognizing and treating them. However, similar findings have not yet been reported in the United States, and specific health services more commonly used by children later diagnosed with ASD and ADHD have not yet been identified.

Based on known early risk factors for both disorders, distinctive patterns of utilization are likely to be present in the electronic health record (EHR) from an early age. Both ASD and ADHD are associated with lower birth weight^23–25^, preterm birth, low APGAR scores, and other perinatal complications^26,27^ that may lead to procedures and increase patients’ length of stay. Early ASD-related comorbidities^28^, such as postnatal hyperbilirubinemia^29^ and respiratory infections^30^, also appear in the EHR as possible indicators of increased risk. Problems with crying, sleeping, and feeding more common in infants with ADHD^31^ and ASD^32^ may lead parents to seek additional support, leading to higher use of specific health services. Utilization patterns may be unique among children with co-occurring ASD and ADHD, who are often diagnosed with ASD much later than children without ADHD symptoms^33,34^. While some risk factors for ASD and ADHD are based on physiologic or diagnostic findings that are not always available, others relate to health interactions readily accessed through most EHR systems.

Identifying EHR-based risk factors is a critical step toward earlier referral, diagnosis, and treatment, which in turn lead to markedly improved outcomes in both ASD^35–40^ and ADHD^41–43^ and reduced healthcare costs^44^. Multiple studies have illustrated the potential of EHR-based prediction models^45^, which can utilize existing EHR data standards to analyze a very large number of predictors^46^. This approach can also uncover unknown risk factors to further illuminate the early trajectories and biological bases of neurodevelopmental disorders and other pediatric conditions. Unlike data from Medicaid claims or health maintenance organization registries^19,47^, EHR data can be immediately analyzed within the hospital system itself to inform provider decision-making during routine care.

In this study, we retrospectively analyzed EHR data collected over a 12.5-year period in a large U.S. health system to compare health system utilization before age 1 between children later diagnosed with ASD and/or ADHD and other children. Analysis focused on hospital admissions, emergency department (ED) visits, outpatient clinic encounters, and procedures across multiple health services. We hypothesized that (a) children later diagnosed ASD and/or ADHD interacted with the health system at higher rates, including increased rates of admissions, ED visits, and non-routine procedures; (b) these children visited specific medical specialties at higher rates, including neurology and gastroenterology in ASD^48–50^; and (c) distinct patterns of interactions were present in each disorder. Our primary aim was to improve our understanding of the early trajectories of children with ASD and ADHD, contributing to earlier, EHR-based risk stratification. A secondary aim was to illustrate a framework that could be used to analyze early healthcare trajectories in other pediatric conditions.

## Patients and methods

### Cohort identification and data extraction

All results are based on retrospective data analysis approved by the Duke Health Institutional Review Board and conducted at the Duke University School of Medicine (2/1/2019–10/1/2019). Analyses were executed within the Duke Protected Analytics Computing Environment (PACE), a highly protected virtual network space designed for protected health information. Participant consent was waived due to the minimal risk posed by study procedures and infeasibility of obtaining consent in a large retrospective cohort.

Analyses were based on inpatient and outpatient encounters within the Duke University Health System (DUHS), a large academic medical center based in Durham, North Carolina. DUHS is comprised of Duke University Hospital, a large (957 bed) facility that includes a regional emergency/trauma center and major (51 operating rooms) surgery suite; Duke Regional Hospital, a medium (369 bed) facility serving Durham and 5 surrounding counties; Duke Raleigh Hospital, a medium (189 bed) facility serving Raleigh and Wake County; and a large network of outpatient facilities, including 30 Duke Primary Care locations. The surrounding region, called the Research Triangle, includes the cities of Raleigh, Durham, and Chapel Hill, and has an estimated population of 2,079,687 (2019 census). Records were extracted from the current (2013—present) DUHS EHR, which is based on the platform developed by Epic (Verona, Wisconsin), as well as several EHR platforms operating prior to 2013.

Study inclusion criteria were (1) date of birth between 10/1/2006 and 10/1/2016; and (2) ≥ 2 outpatient well-child visits within the DUHS before age 1. Criterion (2) was designed to limit analyses to patients likely to have received routine care through DUHS before age 1. Well-child visits were identified using visit type fields present in current and legacy EHR systems. DUHS encounters between 10/1/2006 and 2/1/2019 were extracted for patients meeting criteria. Children diagnosed with ASD or ADHD before age 1 were excluded to ensure that observed health system interactions were not a consequence of an existing diagnosis.

### Diagnosis identification

Five mutually exclusive diagnosis groups were defined based on billing codes from the International Classification of Diseases, Ninth Revision, Clinical Modification (ICD-9-CM) and Tenth Revision, Clinical Modification (ICD-10-CM). Problem list-based diagnoses were excluded due to concerns regarding data quality and availability.

Patients with an ASD or ADHD diagnosis were grouped as ASD (i.e., ASD only), ADHD (i.e., ADHD only), or ASD + ADHD. ASD was defined as ICD-9-CM code 299.00, 299.80, or 299.90^51^; or ICD-10-CM code F84.0, F84.8, or F84.9. ADHD was defined as ICD-9-CM code 314.0, 314.01, 314.1, 314.2, 314.8, or 314.9; or ICD-10-CM code F90.0, F90.1, F90.2, F90.8, or F90.9. Code selection was guided by literature review and the authors’ knowledge of billing practices.

Patients without an ASD or ADHD diagnosis were grouped as upper respiratory infection (URI) or No Diagnosis. URI was chosen to serve as a comparison group due to its status as a common but non-chronic diagnosis. URI was defined as any ICD-9-CM or ICD-10-CM code categorized as URI by Clinical Classification Software (CCS) developed by the Agency for Healthcare Research and Quality^52^.

### Data preprocessing

Analyses focused on rates of hospital admissions, outpatient clinic visits, ED encounters, and procedures among patients in the five previously defined groups. Hospital admissions were further categorized by discharge service rather than admission service, which was not reported prior to 2013. Similarly, outpatient clinic visits were further categorized by clinic service or specialty. Discharge and clinic services were grouped into clinically meaningful and interpretable service types (eTables 8–9). ED encounters resulting in a hospital admission were counted separately from the admission itself. Procedures were categorized by applying CCS to the associated ICD-9-CM or ICD-10-CM code, then further grouping related CCS categories (eTable 10). The following CCS categories were grouped as routine procedures and omitted from the analysis: “Other diagnostic and therapeutic procedures”, “Vaccinations and inoculations”, and “Circumcision”.

Birth encounters (admission source was “born in hospital” or admission date matched date of birth) were analyzed separately from other admissions. Encounter length was calculated as the time difference between arrival and discharge (non-birth encounters) or birth and discharge (birth encounters).

Medicaid status was determined based on documented insurance status associated with all encounters before age 1. Patients whose status was most commonly documented as “Medicaid”, “NC Medicaid”, or “Medicaid Pending” were grouped as “Medicaid”. This group includes patients receiving assistance through the Innovations Waiver program offered to children with documented developmental disabilities by the Division of Mental Health, Developmental Disabilities and Substance Abuse Services within the North Carolina Department of Health and Human Services. Patients whose insurance status was not documented in any encounter were grouped as “Unknown”. Other patients were grouped as “Non-Medicaid”.

### Statistical analysis

Group differences in demographic variables (sex, race, ethnicity, Medicaid status) were assessed via chi-square test. Encounters and procedures before age 1 in each category described previously were counted for each patient. Group differences in encounters and procedures were then assessed by (a) comparing counts by pairwise two-tailed Mann–Whitney *U* test, and (b) comparing the proportion of patients with at least one occurrence (i.e. rate of occurrence) by two-tailed two-sample proportion test. Differences in length of stay were assessed by two-tailed Mann–Whitney *U* test. Mann–Whitney *U* tests (rather than parametric tests) were selected because count and length of stay data are non-negative and long-tailed.

Raw p-values have been reported throughout, but statistical significance was determined by applying Bonferroni correction with an initial threshold of $\alpha =0.05$. Results with $p<\alpha /m$, where $m$ is the number of categories for a given analysis, were considered statistically significant. In the analyses of outpatient clinic services, for example, 9 distinct types of services were examined, therefore results with *p* < 0.0056 were considered statistically significant.

Logistic regression was used to control for sex, race (White, Black or African American, Asian, Other Race), ethnicity (Hispanic or Latino, not Hispanic or Latino), and Medicaid status when comparing encounter and procedure rates between groups. Models were implemented in Statsmodels v0.11^53^ with Python 3.6^54^. An additional binary variable (Epic, not Epic) was included to control for differences in encounter and procedure reporting between the current, Epic-based EHR platform and legacy platforms prior to 2013. Two-tailed t-tests were applied to all model parameters, and those with $p<\alpha /m$ (as above) were considered statistically significant. Corresponding confidence intervals and adjusted odds ratios (AOR) were based on the Student’s t-distribution.

### Institutional review and informed consent

All methods were carried out in accordance with the relevant guidelines and regulations. Study procedures were approved by the Duke Health Institutional Review Board and comply with institutional policies and federal regulations. A waiver of participant consent was approved due to the minimal risk posed by study procedures and infeasibility of obtaining consent in a large retrospective cohort. Analyses were executed within the Duke Protected Analytics Computing Environment (PACE), a highly protected virtual network space designed for protected health information.

## Results

### Description of cohort

Records for 200,423 unique patients born 10/1/2006–10/1/2016 were initially extracted, and 29,931 had ≥ 2 well-child visits before age 1. T wo patients were excluded due to ADHD diagnosis before age 1, leaving 29,929 patients who met study criteria. Demographics by group and age at diagnosis are presented in Table 1.

**Table 1:** Participant demographics and age at diagnosis.

Variable	Category/Value	All	No Dx	ASD	ADHD	ASD+ADHD		URI
Total N (%)		29,929	20,387 (68.1%)	343 (1.1%)	1175 (3.9%)	140 (0.5%)		7884 (26.3%)
Sex, N (%)	Male	15,425 (51.5%)	10,351 (50.8%)	268 (78.1%)	836 (71.1%)	123 (87.9%)		3847 (48.8%)
	Female	14,504 (48.5%)	10,036 (49.2%)	75 (21.9%)	339 (28.9%)	17 (12.1%)		4037 (51.2%)
	*χ*^*2*^ (*df* = 1)		14.9	97.2	187.5	72.8		32.1
	*p* value		< 0.001	< 0.001	< 0.001	< 0.001		< 0.001
Race, N (%)	White	12,692 (45.8%)	8552 (41.9%)	119 (38.1%)	536 (46.9%)	59 (43.4%)		3426 (46.3%)
	Black or African American	9475 (34.2%)	6309 (30.9%)	120 (38.5%)	487 (42.6%)	56 (41.2%)		2503 (33.8%)
	Not reported	1884 (6.8%)	1321 (6.5%)	20 (6.4%)	49 (4.3%)	6 (4.4%)		488 (6.6%)
	Two or more races	1964 (7.1%)	1341 (6.6%)	31 (9.9%)	59 (5.2%)	13 (9.6%)		520 (7.0%)
	Asian	1548 (5.6%)	1088 (5.3%)	19 (6.1%)	8 (0.7%)	2 (1.5%)		431 (5.8%)
	American Indian or Alaska Native	108 (0.4%)	86 (0.4%)	2 (0.6%)	2 (0.2%)	0 (0.0%)		18 (0.2%)
	Native Hawaiian or other Pacific Islander	50 (0.2%)	35 (0.2%)	1 (0.3%)	1 (0.1%)	0 (0.0%)		13 (0.2%)
	*χ*^*2*^ (*df* = 6)		63.3	10.6	133.6	14.7		23.4
	*p* value		< 0.001	0.102	< 0.001	0.023		0.001
Ethnicity, N (%)	Not hispanic/latino	24,540 (82.0%)	16,573 (81.3%)	272 (79.3%)	1043 (88.8%)	116 (83.5%)		6536 (82.9%)
	Hispanic/latino	3497 (11.7%)	2487 (12.2%)	51 (14.9%)	83 (7.1%)	17 (12.2%)		859 (10.9%)
	Not reported	1884 (6.3%)	1321 (6.5%)	20 (5.8%)	49 (4.2%)	6 (4.3%)		488 (6.2%)
	*χ*^*2*^ (*df* = 2)		21.8	3.4	37.9	0.9		7.0
	*p* value		< 0.001	0.179	< 0.001	0.625		0.031
Medicaid Status, N (%)	Medicaid	6227 (20.8%)	4604 (19.9%)	100 (29.2%)	69 (5.9%)	16 (11.4%)		1438 (18.2%)
	Non-Medicaid	8488 (28.4%)	6024 (29.5%)	89 (25.9%)	45 (3.8%)	18 (12.9%)		2312 (29.3%)
	Unknown	15,214 (50.8%)	9759 (47.9%)	154 (44.9%)	1061 (90.3%)	106 (75.7%)		4134 (52.4%)
			239.5	14.7	765.3	35.0		42.8
			< 0.001	< 0.001	< 0.001	< 0.001		< 0.001

				ASD	ADHD	ASD	ADHD	URI
Diagnosis Age, in years	Mean ± SD			4.1±1.8	7.2±1.8	5.5±2.4	5.9±1.8	2.4±1.7
	Range (min, max)			(1.3, 10.5)	(1.0, 12.6)	(1.5, 12.1)	(1.8, 12.3)	(1.0, 12.5)

*ASD* Autism spectrum disorder, *ADHD *attention deficit hyperactivity disorder, *ASD + ADHD* co-occurring ASD and ADHD, *URI* upper respiratory infection, *No Dx* No ASD, ADHD, or URI diagnosis.

Among patients meeting criteria, unadjusted ASD and ADHD prevalence were 1.6% and 4.4%, respectively. Co-occurring ASD + ADHD (N = 140) was identified in 29.0% of patients with ASD and 10.6% of patients with ADHD. Mean age at ASD diagnosis was 1.4 years higher in patients with co-occurring ADHD (5.5y vs. 4.1y), whereas mean age at ADHD diagnosis was 1.3 years lower in patients with co-occurring ASD (5.9y and 7.2y).

Male to female ratios were 3.6 for ASD, 2.5 for ADHD, and 7.2 for ASD + ADHD. ADHD diagnosis was strongly associated with racial (*Χ*^2^ = 133.6, *dof* = 6, *p* < 0.001) and ethnic status (*Χ*^2^ = 37.9, *dof* = 2, *p* < 0.001), but ASD diagnosis was not (*Χ*^2^ = 10.6, *dof* = 6, *p* = 0.102; and *Χ*^2^ = 3.4, *dof* = 2, *p* = 0.179, respectively). Both diagnoses were associated with Medicaid status (ASD: *Χ*^2^ = 14.7, *dof* = 2, *p* < 0.001; ADHD *Χ*^2^ = 765.3, *dof* = 2, *p* < 0.001). Among patients with known insurance status, Medicaid rates were 52.9% and 60.5% in the ASD and ADHD groups, respectively, compared to 42.3% in the No Diagnosis group.

### Summary of health system utilization

**Figure 1:**
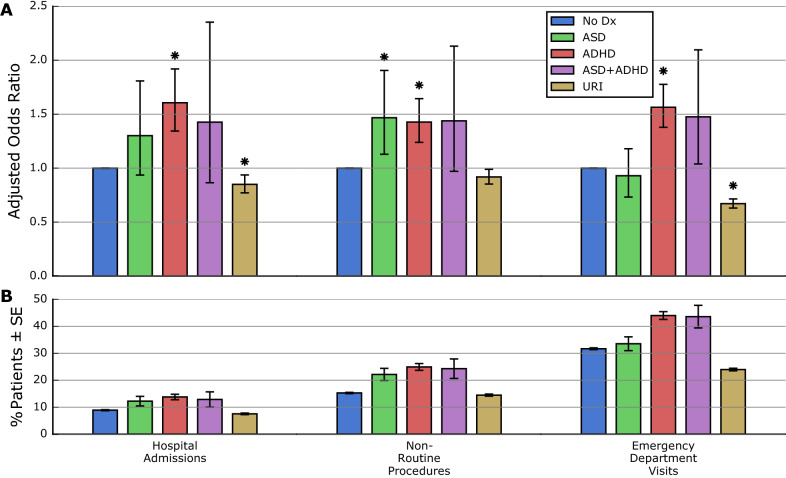
Adjusted odds ratios and rates of hospital admissions, procedures, and ED visits. Adjusted odds ratios (AORs) (**A**) and unadjusted occurrence rates (**B**) in each diagnosis group are shown for non-birth hospital admissions, procedures, and emergency department visits. Error bars indicate 95% confidence intervals for the AOR (**A**) and the standard error of the proportion (**B**). AORs were assessed for statistical significance (*) after applying Bonferroni correction to a baseline threshold of *α* = 0.05. Unadjusted occurrence rates are descriptive only, and were not tested for statistical significance.

Most patients (87.5%) were born within DUHS. Before age 1, 8.8% were admitted (non-birth) to a DUHS hospital, 8.8% underwent a procedure, and 30.2% visited an ED. Hospital admission and procedures were more likely in ASD (AOR = 1.30, *p* = 0.1173; AOR = 1.47, *p* = 0.0042), although only the latter reached statistical significance, and in ADHD (AOR = 1.61, *p* < 0.0001; AOR = 1.43, *p* < 0.0001). In contrast, ED visits were more likely in ADHD (AOR = 1.56, *p* < 0.0001), but not in ASD (AOR = 0.93, *p* = 0.5454). Hospital admission, procedures, and ED visits were increased in ASD + ADHD but not statistically significant due to smaller group size (AOR = 1.43, *p* = 0.1643; AOR = 1.44, *p* = 0.0703; AOR = 1.48, *p* = 0.0301). These rates were not increased in URI (Fig. 1, eTable 1).

Hospital admission and ED visits were more likely in males (AOR = 1.13, *p* = 0.0034; AOR = 1.13, *p* < 0.0001), but procedures were not (AOR = 1.08, *p* = 0.0237). Rates of hospital admissions, procedures, and ED visits were also higher among African American, Hispanic/Latino, and Medicaid patients and lower among White and Asian patients (eTable 2).

All patients had ≥ 2 outpatient clinic encounters for inclusion, but the mean number of these encounters was higher in ASD (14.8, *p* < 0.001), ADHD (14.7, *p* < 0.001), and ASD + ADHD (14.6, *p* < 0.001) compared to No Diagnosis (12.5), whereas it was lower in URI (11.4, *p* < 0.001).

### Outpatient clinic services

**Figure 2:**
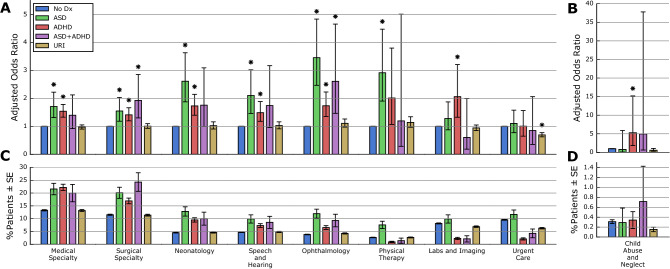
Adjusted odds ratios and rates of hospital admissions by discharge service category. Adjusted odds ratios (AORs) (**A**,**B**) and unadjusted occurrence rates (**C**,**D**) are shown for nine outpatient clinic visit types (see eTable 8 for definitions). Error bars indicate 95% confidence intervals for the AOR (**A**,**B**) and the standard error of the proportion (**C**,**D**). AORs were assessed for statistical significance (*) after applying Bonferroni correction to a baseline threshold of *α* = 0.05. Unadjusted occurrence rates are descriptive only, and were not tested for statistical significance. Child abuse and neglect (**B**,**D**) has been plotted with a different y-scale due to its lower rate of occurrence.

Distinct patterns of outpatient clinic encounters were observed in ASD versus ADHD. Visits to medical and surgical specialists were increased (Fig. 2, eTable 1), with the latter being even more likely in ASD + ADHD (AOR = 1.93, *p* < 0.0011). ASD and ADHD groups were both more likely to visit neonatology (AOR = 2.61, *p* < 0.0001; AOR = 1.73, *p* < 0.0001), speech pathology (AOR = 2.52, *p* < 0.0001; AOR = 1.45, *p* = 0.0033), and ophthalmology (AOR = 3.45, *p* < 0.0001; AOR = 1.74, *p* < 0.0001), but these increases were more pronounced in ASD.

Adjusted odds of physical therapy were increased almost threefold in ASD (AOR = 2.92, *p* < 0.0001) and twofold in ADHD (AOR = 2.01, *p* = 0.0306), although only the former was statistically significant. In contrast, adjusted odds of visits related to child abuse and neglect were increased over fivefold in ADHD only (AOR = 5.30, *p* = 0.0019). Adjusted odds of diagnostic visits (*e.g.*, labs, radiology) were also higher in ADHD (AOR = 2.06, *p* = 0.0014).

The allocation of visits among medical and surgical specialties differed between groups (eFigure 1). Among medical specialties, cardiology and medicine visits were more common in ADHD (AOR = 1.53, *p* = 0.0004; AOR = 1.47, *p* = 0.0008), whereas endocrinology, gastroenterology, and neurology visits were over threefold more likely in ASD (AOR = 3.39, *p* < 0.0001; AOR = 3.25, *p* = 0.0001, AOR = 3.15, *p* < 0.0001) (eFigure 2, eTable 3). Among surgical specialties, general surgery visits were most common in ADHD (AOR = 1.73, *p* = 0.0006), whereas ear, nose, and throat visits were twice as likely in ASD (AOR = 2.01, *p* = 0.0031) (eFigure 3, eTable 3).

Statistically significant differences between demographic groups included higher rates of general surgery visits among male (AOR = 2.60, *p* < 0.0001), African American (AOR = 1.52, *p* < 0.0001), and Medicaid (AOR = 2.34, *p* < 0.0001) patients, higher rates of cardiology visits among African American (AOR = 1.41, *p* = 0.0003), Hispanic/Latino (AOR = 1.46, *p* = 0.0003), and Medicaid (AOR = 1.46, *p* = 0.0034) patients, and higher rates of audiology (AOR = 1.79, *p* < 0.0001) and speech pathology visits (AOR = 1.94, *p* < 0.0001) among Hispanic/Latino patients (eTable 4). A number of visit types were substantially more likely in Medicaid patients, including physical therapy (AOR = 79.86, *p* < 0.0001), labs and imaging (AOR = 38.58, *p* < 0.0001), and urgent care (AOR = 84.13, *p* < 0.0001).

### Procedures

**Figure 3:**
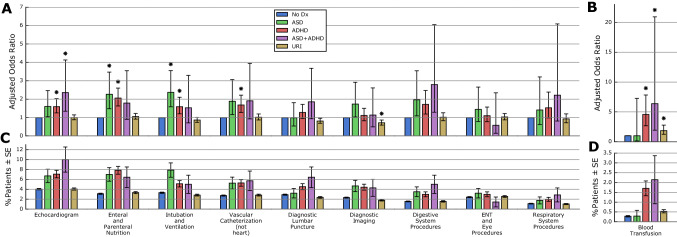
Adjusted odds ratios and rates of procedures. Adjusted odds ratios (AORs) (**A**,**B**) and unadjusted occurrence rates (**C**,**D**) are shown for ten categories (see eTable 10 for definitions) of procedures from both birth and non-birth encounters. Error bars indicate 95% confidence intervals for the AOR (**A**,**B**) and the standard error of the proportion (**C**,**D**). AORs were assessed for statistical significance (*) after applying Bonferroni correction to a baseline threshold of *α* = 0.05. Unadjusted occurrence rates are descriptive only, and were not tested for statistical significance. Blood transfusion (**B**,**D**) has been plotted with a different y-scale due to its lower rate of occurrence.

Distinct patterns of procedure occurrence before age 1 were also observed in ASD versus ADHD (Fig. 3, eTable 1). Echocardiograms were more likely in ASD (AOR = 1.60, *p* = 0.0327), although not statistically significant; ADHD (AOR = 1.59, *p* = 0.0002); and ASD + ADHD (AOR = 2.36, *p* = 0.0028). Enteral and parental nutrition were also more likely in all three groups, although not statistically significant in ASD + ADHD (ASD AOR = 2.26, *p* = 0.0002; ADHD AOR = 2.06, *p* < 0.0001; ASD + ADHD AOR = 1.79, *p* = 0.0966).

Intubation and ventilation were twice as likely in ASD (AOR = 2.37, *p* < 0.0001), but adjusted odds were also increased in ADHD (AOR = 1.59, *p* = 0.0012). Blood transfusions were over four times more likely in ADHD (AOR = 4.57, *p* < 0.0001) and in ASD + ADHD (AOR = 6.38, *p* = 0.0022), but not in ASD (AOR = 1.00, *p* = 0.9989).

Procedures from birth versus non-birth encounters were also analyzed independently (eTables 5–6).

### Admission services and length of stay

Patients were discharged by Pediatrics in 79.7% of all non-birth hospital admissions, therefore adjusted odds of discharge by Pediatrics (eFigure 4) are similar to hospital admission rates (Fig. 1). Whereas higher rates of hospital discharge by Pediatrics were observed in ASD (AOR = 1.51, *p* = 0.0171) and statistically significant in ADHD (AOR = 1.57, *p* < 0.0001), higher rates of discharge by Neonatology were found only in ADHD (AOR = 2.06, *p* = 0.0014), and in ASD + ADHD (AOR = 2.12, *p* = 0.2025).

**Figure 4:**
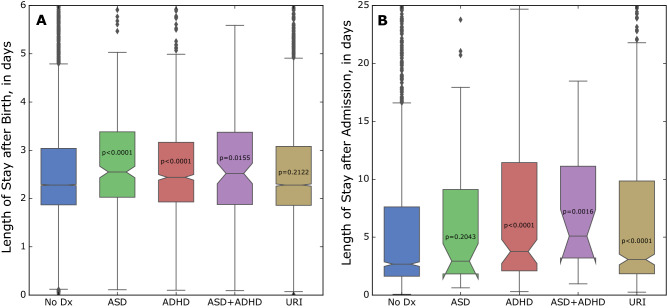
Length of Hospital Stay by Diagnosis. (**A**) shows the length of hospital stay after birth for patients in each diagnosis group. (**B**) shows the length of hospital stay after non-birth admission for patients in each diagnosis group. Box midlines and edges show the median and interquartile range (IQR), respectively. The notches indicate a 95% confidence interval for the median, which can extend beyond the IQR (e.g. ASD and ASD + ADHD in **B**). Lower and upper whiskers extend to outliers within $1.5*\mathrm{IQR}$ of first and third quartile, respectively. Outliers have been clipped to show boxplot detail, but were included in the statistical analysis.

The typical hospital stay (non-birth admission) was over a day longer in ADHD (median = 3.76d, *p* < 0.0001) and over two days longer in ASD + ADHD (median = 5.09d, *p* = 0.0032) compared to those in the No Diagnosis group (median = 2.67d). The typical hospital stay after birth was 6.5 h longer in ASD (median = 2.55d, *p* < 0.0001), 3.8 h longer in ADHD (median = 2.44d, *p* < 0.0001), and 5.8 h longer in ASD + ADHD (median = 2.52d, *p* = 0.0310) compared to those in the No Diagnosis group (median = 2.28 days) (Fig. 4, eTable 7).

## Discussion

This is the first study reporting health system utilization among individuals with ASD, ADHD, and co-occurring ASD + ADHD in their first year of life, prior to diagnosis. Analysis drew on records from over 200,000 unique patients visiting DUHS over a 12.5-year period, almost 30,000 of whom met study criteria. Results corroborate studies from Europe demonstrating increased utilization prior to ADHD diagnosis, extend this finding to ASD and co-occurring ASD + ADHD, and highlight distinct interaction patterns underlying the increased rates of utilization observed in both disorders. We also highlight the demographics of co-occurring ASD + ADHD, which was associated with a 1.4 year delay in ASD diagnosis^33,34^ and a higher male to female ratio (7.2) than either disorder alone. More broadly, this work demonstrates that ASD and ADHD have profound effects on health and health system resources prior to diagnosis. Thus, EHR platforms provide an opportunity to monitor health interactions early in life to stratify patients’ risk of developing ASD and ADHD, and may offer a unique window into the early trajectory and biological bases of these conditions.

Many of these findings are not surprising given current knowledge of characteristics and comorbidities associated with ASD and ADHD. Higher rates of clinic follow-up, hospital admission, and intubation may result from the association with perinatal complications found in both disorders^26,27^, for example, but our results show that these associations have measurable impact on a health system level. Additionally, many other findings were unexpected. In ASD, increased visits to gastroenterology and neurology were expected, but increased visits to ophthalmology and endocrinology were not; this finding suggests that these comorbidities^28^ also have substantial impact on early utilization. In ADHD, increased odds of blood transfusion and visits related to child abuse and neglect are consistent with known associations with ADHD^55^, including problems in infancy^31^ and increased parental stress previously observed in infants with ADHD^12^, but are striking nevertheless.

The size and number of statistically significant effects suggest that early health interactions contain valuable information about ASD and ADHD risk, implying that health services data from the EHR could inform risk stratification. Given the importance of prompt ASD and ADHD diagnosis, further work is needed to determine how this information might be leveraged to inform clinician decision-making and/or health system policy. Risk monitoring systems could be implemented within the EHR itself, for example, in order to automatically alert the families and/or providers of children whose ASD or ADHD risk is high. Follow-up work should not only implement and validate risk monitoring, but also explore the effectiveness of a range of interventions aimed at families, individual providers, and the health system as a whole. Moreover, this paradigm could be applied to promote earlier identification and intervention in a wide range of pediatric conditions.

The current findings demonstrate that patterns of health system utilization associated with ASD and ADHD, such as longer hospital stays after birth, emerge in the first few days of life, and many more emerge within the first year. Increases in specific forms of utilization, such as ED visits, are not limited to ASD or ADHD, therefore additional work is needed to determine which utilization patterns—or combinations of patterns—have high predictive value. Subsequent work will focus on the development of predictive models that can be deployed and iteratively evaluated in clinical workflow.

### Limitations

Results are observational and associative, and should be interpreted with a degree of caution due to possible confounding effects. Analyses controlled for sex, race, ethnicity, insurance status, and an institutional transition between EHR systems that affected the reporting of some encounter types. Socioeconomic status and other potentially relevant covariates, such as family characteristics, were not consistently available. Importantly, our analysis did account for a potentially impactful confounder, namely, the presence of a diagnosis after age 1. By including the URI comparison group, we demonstrate that selecting children with a non-chronic diagnosis who remain engaged with the health system does not produce similar effects to those observed in ASD and ADHD. Future work should focus on comparisons to other chronic health conditions (e.g*.*, asthma, congenital heart disease), and on understanding differences between demographic groups, including disadvantaged groups, in greater detail.

Analysis was based on all patients born 10/1/2006–10/1/2016 who received routine care within DUHS prior to age 1. Our observation window (10/1/2006–2/1/2019) implies that patients’ ages ranged from 2.3 to 12.3 years at the end of the observation. Patients in our cohort may have been (or still be) diagnosed with ASD or ADHD subsequent to observation, or at facilities outside of DUHS. Thus, analyses should be viewed as comparing patients with known ASD and ADHD diagnoses to all other patients, including those with other medical and psychiatric conditions, rather than a cohort of typically developing children. Results demonstrate that children later diagnosed with ASD and ADHD utilize health system resources at higher rates than the average child.

EHR-based analyses are affected by known data quality issues, including unreliable race and ethnicity reporting. Our analysis groups do not reflect gold standard diagnoses, and identification of ADHD by diagnosis codes was 70–75% accurate across 10 institutions^56^. Further, analysis was based on DUHS encounters only, whereas patients may have received additional care outside of DUHS. Study criteria (e.g*.*, ≥ 2 well-child visits) were designed to identify patients receiving routine care within DUHS, but their effectiveness is uncertain. These limitations are consistent with our overarching goal to identify EHR-based predictors of ASD and ADHD, but results may not generalize to health systems and/or geographic regions with different demographic makeup compared to DUHS and its surrounding area; or to other sources of health utilization data, particularly non-EHR data sources.

## Conclusion

ASD and ADHD are associated with increased health system utilization in the first year of life, prior to diagnosis. Moreover, the two disorders are associated with distinct patterns of early health interactions that could be monitored through the EHR to stratify patients’ risk of developing ASD and ADHD. Subsequent work will focus on developing predictive models that could be deployed within a health system to inform provider decision-making, contributing to earlier diagnosis and treatment.

## Supplementary information

Supplementary information

## Abbreviations

ASD: Autism spectrum disorder
ADHD: Attention deficit hyperactivity disorder
URI: Upper respiratory infection
ED: Emergency department
AOR: Adjusted odds ratio
EHR: Electronic health records
DUHS: Duke University Health System
CCS: Clinical classification software
