# Supplementary material for: Health system utilization before age 1 among children later diagnosed with autism or ADHD
Source: Sci Rep. 2020 Oct 19;10:17677. doi: 10.1038/s41598-020-74458-2 (PMC7572401; doi:10.1038/s41598-020-74458-2)
Supplement: Supplementary file 1 — Supplementary information [file 41598_2020_74458_MOESM1_ESM.docx]

**Health System Utilization before Age 1 among Children Later Diagnosed with Autism or ADHD**

^1^Matthew M Engelhard, MD, PhD, ^2,3^Samuel I Berchuck, PhD, ^4^Jyotsna Garg, MS, ^3,4,5^Ricardo Henao, PhD, ^3^Andrew Olson, MPP, ^3^Shelley Rusincovitch, MMCi, ^1,6^Geraldine Dawson, PhD, ^1,4^Scott H Kollins, PhD

^1^Department of Psychiatry and Behavioral Sciences, Duke University School of Medicine

^2^Department of Statistical Science, Duke University

^3^Duke Forge, Duke University School of Medicine

^4^Duke Clinical Research Institute, Duke University School of Medicine

^5^Department of Biostatistics and Bioinformatics, Duke University School of Medicine

^6^Duke Center for Autism and Brain Development and Duke Institute for Brain Sciences

**Supplementary Content:**

**eFigure 1:** Allocation of Specialty Care by Diagnosis

**eFigure 2:** Adjusted Odds Ratios and Rates of Outpatient Medical Specialty Care

**eFigure 3:** Adjusted Odds Ratios and Rates of Outpatient Surgical Specialty Care

**eFigure 4:** Adjusted Odds Ratios and Rates of Non-Birth Hospital Admissions by Discharge Service Category

**eTable 1:** Adjusted Odds Ratios and Rates of Non-Birth Hospital Admissions, Procedures, Emergency Department Visits, and Outpatient Clinic Services by Diagnosis

**eTable 2:** Adjusted Odds Ratios for Non-Birth Hospital Admissions, Procedures, Emergency Department Visits, and Outpatient Clinic Services by Demographic Factors

**eTable 3:** Adjusted Odds Ratios and Rates of Outpatient Specialty Care by Diagnosis

**eTable 4:** Adjusted Odds Ratios for Outpatient Specialty Care by Demographic Factors

**eTable 5:** Adjusted Odds Ratios and Rates of Procedures from Birth and Non-Birth Encounters by Diagnosis

**eTable 6:** Adjusted Odds Ratios for Procedures from Birth and Non-Birth Encounters by Demographic Factors

**eTable 7:** Length of Hospital Stay after Birth and after Non-Birth Admissions by Diagnosis

**eTable 8:** Group Definitions for Outpatient Clinic Encounters

**eTable 9:** Group Definitions for Inpatient Services

**eTable 10:** Group Definitions for Procedures

**eFigure 1: Allocation of Specialty Care by Diagnosis**. The charts show how visits among participants in each group were allocated between medical specialists, surgical specialists, and other specialty care.


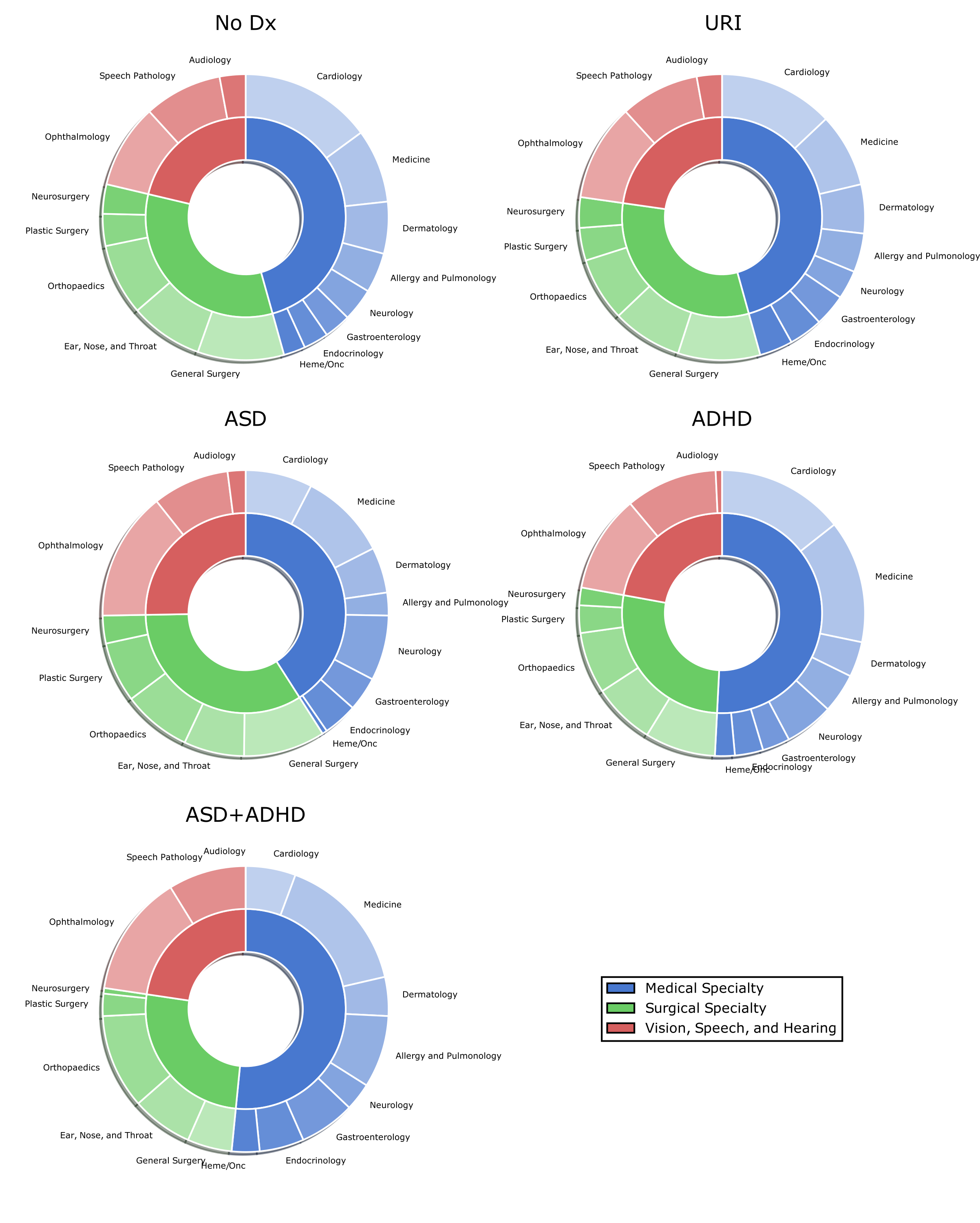


**eFigure 2: Adjusted Odds Ratios and Rates of Outpatient Medical Specialty Care.** Adjusted odds ratios (AORs) (top) and unadjusted occurrence rates (bottom) are shown for outpatient clinic visits to receive medical specialty care. Error bars indicate 95% confidence intervals for the AOR (top) and the standard error of the proportion (bottom). AORs were assessed for statistical significance (*) after applying Bonferroni correction to a baseline threshold of *α=*0.05. Unadjusted occurrence rates are descriptive only, and were not tested for statistical significance.

**
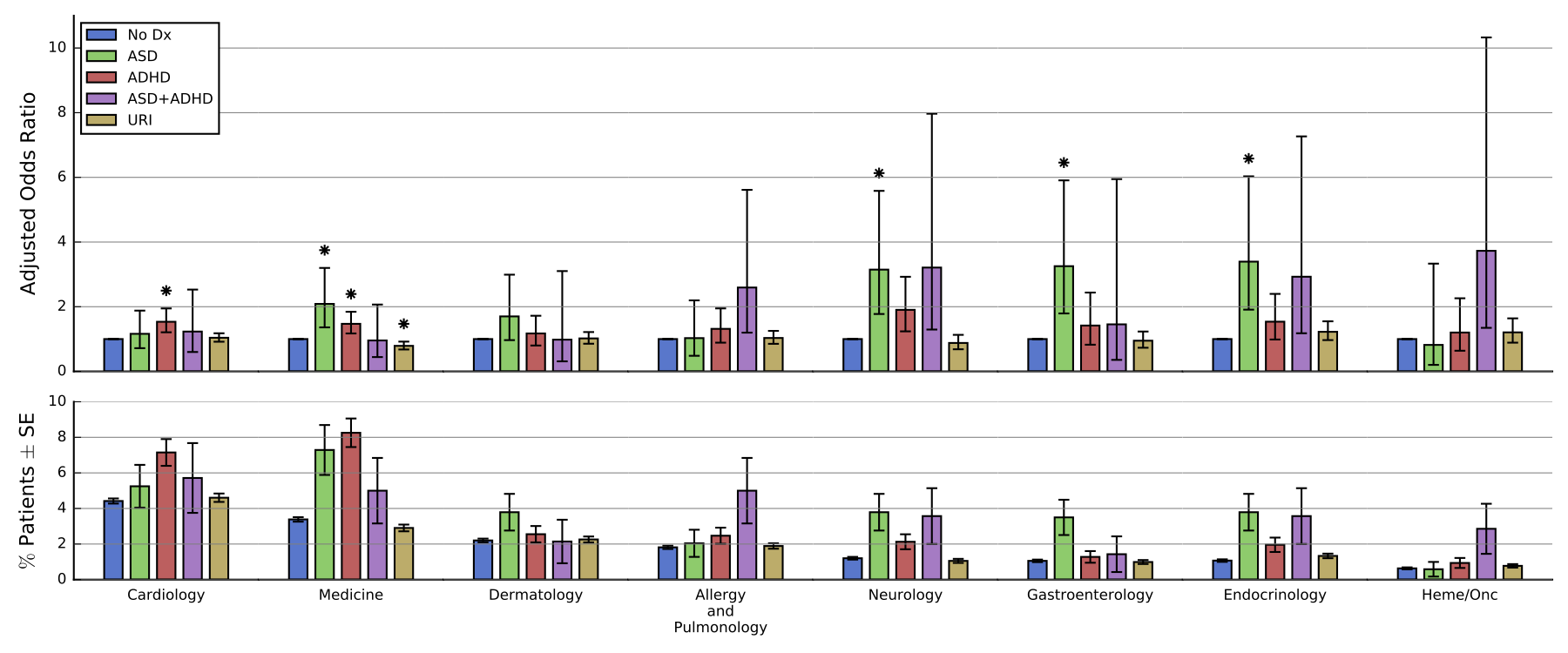
**

**eFigure 3: Adjusted Odds Ratios and Rates of Outpatient Surgical Specialty Care.** Adjusted odds ratios (AORs) (top) and unadjusted occurrence rates (bottom) are shown for outpatient clinic visits to receive surgical specialty care. Error bars indicate 95% confidence intervals for the AOR (top) and the standard error of the proportion (bottom). AORs were assessed for statistical significance (*) after applying Bonferroni correction to a baseline threshold of *α=*0.05. Unadjusted occurrence rates are descriptive only, and were not tested for statistical significance.

**
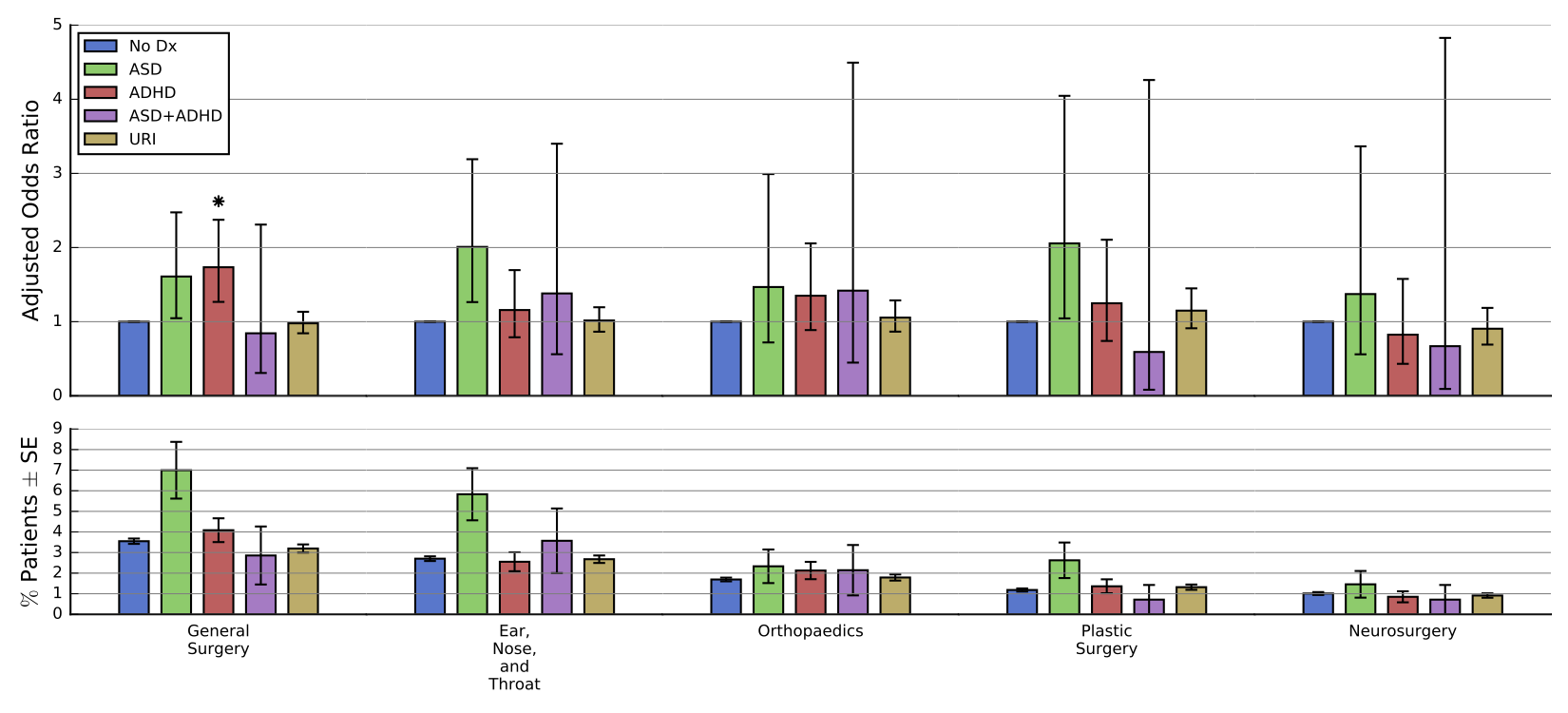
**

**eFigure 4: Adjusted Odds Ratios and Rates of Hospital Admissions by Discharge Service Category.** Adjusted odds ratios (AORs) (top) and unadjusted occurrence rates (bottom) are shown for hospital admissions where the discharge service was listed as Pediatrics, Neonatology, a medical specialty, or a surgical specialty, respectively. Error bars indicate 95% confidence intervals for the AOR (top) and the standard error of the proportion (bottom). AORs were assessed for statistical significance (*) after applying Bonferroni correction to a baseline threshold of *α=*0.05. Unadjusted occurrence rates are descriptive only, and were not tested for statistical significance.

**
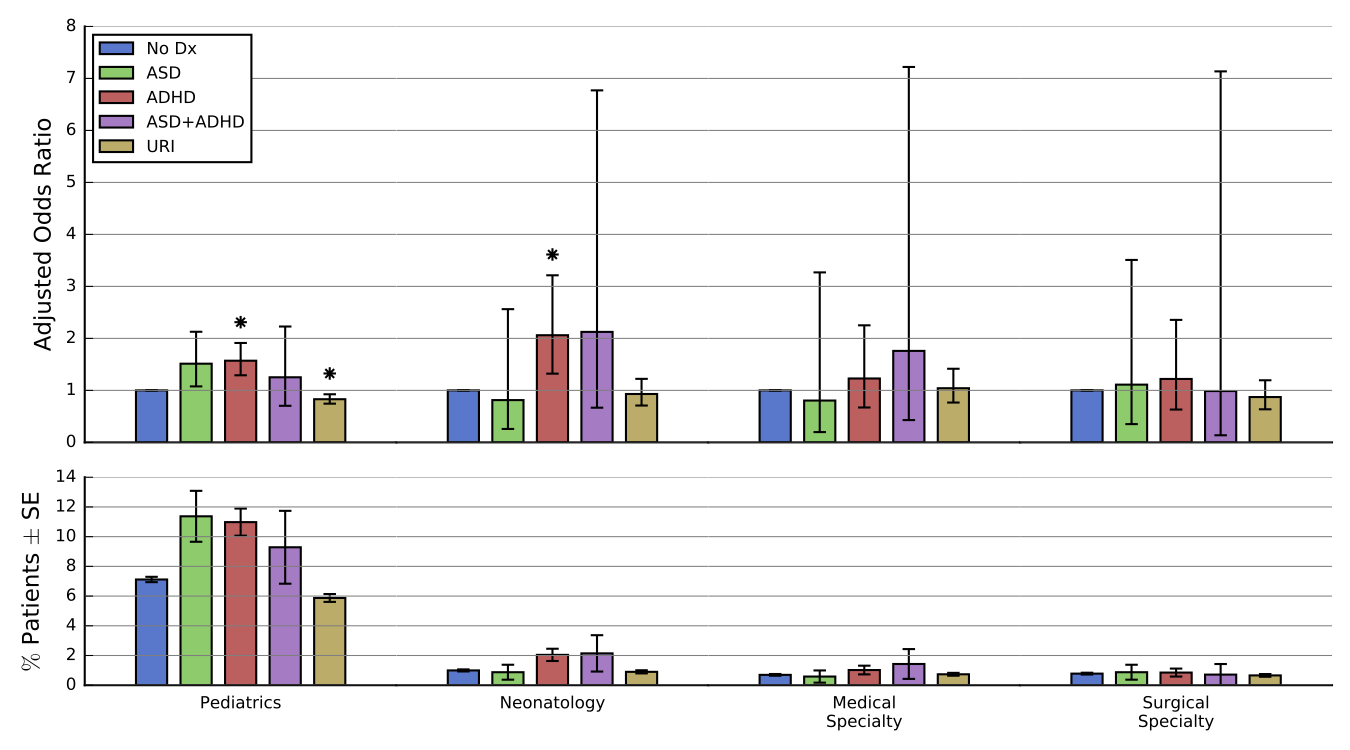
**

**eTable 1:** Adjusted Odds Ratios and Rates of Hospital Admissions, Non-Routine Procedures, Emergency Department Visits, and Outpatient Clinic Services by Diagnosis

|  | **No Dx** | **ASD** | | | **ADHD** | | | **ASD+ADHD** | | | **URI** | | |
| --- | --- | --- | --- | --- | --- | --- | --- | --- | --- | --- | --- | --- | --- |
|  | **%** | **%** | **AOR**  **(95% CI)** | **p** | **%** | **AOR**  **(95% CI)** | **p** | **%** | **AOR**  **(95% CI)** | **p** | **%** | **AOR**  **(95% CI)** | **p** |
| *Hospital Admissions* | 8.9 | 12.2 | 1.30  (0.94-1.81) | 0.1173 | 13.8 | 1.61  (1.34-1.92) | <.0001 | 12.9 | 1.43  (0.86-2.35) | 0.1643 | 7.5 | 0.85  (0.77-0.94) | 0.001 |
| *Non-Routine Procedures* | 15.3 | 22.2 | 1.47  (1.13-1.91) | 0.0042 | 24.9 | 1.43  (1.24-1.64) | <.0001 | 24.3 | 1.44  (0.97-2.13) | 0.0703 | 14.5 | 0.92  (0.85-0.99) | 0.0239 |
| *Emergency Department Visits* | 31.7 | 33.5 | 0.93  (0.73-1.18) | 0.5454 | 44.0 | 1.56  (1.38-1.78) | <.0001 | 43.6 | 1.48  (1.04-2.10) | 0.0301 | 24.0 | 0.67  (0.63-0.71) | <.0001 |
| **Outpatient Clinic Services** | | | | | | | | | | | | | |
| *Medical Specialty* | 13.3 | 21.6 | 1.71  (1.31-2.22) | <.0001 | 22.2 | 1.54  (1.33-1.78) | <.0001 | 20 | 1.40  (0.92-2.12) | 0.1184 | 13.2 | 0.97  (0.90-1.05) | 0.4864 |
| *Surgical Specialty* | 11.5 | 2.1 | 1.55  (1.18-2.03) | 0.0016 | 16.9 | 1.41  (1.20-1.66) | <.0001 | 24.3 | 1.93  (1.30-2.85) | 0.0011 | 11.4 | 1.01  (0.93-1.09) | 0.8625 |
| *Neonatology* | 4.6 | 12.8 | 2.61  (1.88-3.63) | <.0001 | 9.5 | 1.73  (1.40-2.14) | <.0001 | 10 | 1.76  (1.00-3.09) | 0.0511 | 4.6 | 1.02  (0.90-1.16) | 0.7028 |
| *Speech and Hearing* | 4.7 | 9.9 | 2.10  (1.46-3.02) | <.0001 | 7.3 | 1.49  (1.18-1.88) | 0.0009 | 8.6 | 1.74  (0.96-3.17) | 0.0691 | 4.8 | 1.03  (0.91-1.16) | 0.6672 |
| *Ophthalmology* | 3.8 | 12.0 | 3.45  (2.47-4.84) | <.0001 | 6.6 | 1.74  (1.35-2.23) | <.0001 | 9.3 | 2.61  (1.46-4.66) | 0.0012 | 4.3 | 1.11  (0.97-1.26) | 0.1224 |
| *Physical Therapy* | 2.7 | 7.6 | 2.92  (1.90-4.47) | <.0001 | .9 | 2.01  (1.07-3.80) | 0.0306 | 1.4 | 1.19  (0.28-5.01) | 0.8103 | 2.7 | 1.14  (0.97-1.34) | 0.1151 |
| *Labs and Imaging* | 8.2 | 9.9 | 1.28  (0.88-1.87) | 0.2019 | 2.3 | 2.06  (1.32-3.21) | 0.0014 | 2.1 | 0.60  (0.18-1.99) | 0.4078 | 6.9 | 0.94  (0.85-1.05) | 0.2762 |
| *Urgent Care* | 9.6 | 11.7 | 1.10  (0.77-1.58) | 0.5866 | 2.1 | 1.01  (0.65-1.56) | 0.9794 | 4.3 | 0.85  (0.35-2.06) | 0.7224 | 6.3 | 0.69  (0.62-0.77) | <.0001 |
| *Child Abuse and Neglect* | .3 | .3 | 0.81  (0.11-5.88) | 0.8314 | .3 | 5.30  (1.85-15.19) | 0.0019 | .7 | 4.96  (0.65-37.78) | 0.1225 | .2 | 0.58  (0.31-1.08) | 0.0883 |
| **Non-Routine Procedures** | | | | | | | | | | | | | |
| *Echocardiogram* | 4.0 | 6.7 | 1.60  (1.04-2.46) | 0.0327 | 7.1 | 1.59  (1.25-2.03) | 0.0002 | 1.0 | 2.36  (1.34-4.13) | 0.0028 | 4.0 | 1.00  (0.88-1.14) | 0.9986 |
| *Enteral and Parenteral Nutrition* | 3.1 | 7.0 | 2.26  (1.48-3.47) | 0.0002 | 7.8 | 2.06  (1.63-2.60) | <.0001 | 6.4 | 1.79  (0.90-3.55) | 0.0966 | 3.3 | 1.05  (0.91-1.22) | 0.4784 |
| *Intubation and Ventilation* | 3.3 | 7.9 | 2.37  (1.58-3.55) | <.0001 | 5.1 | 1.59  (1.20-2.10) | 0.0012 | 5.0 | 1.53  (0.71-3.29) | 0.2799 | 2.8 | 0.86  (0.74-1.00) | 0.0562 |
| *Vascular Catheterization (not heart)* | 2.7 | 5.2 | 1.88  (1.16-3.06) | 0.0106 | 5.3 | 1.68  (1.27-2.22) | 0.0002 | 5.7 | 1.91  (0.93-3.94) | 0.08 | 2.8 | 1.02  (0.87-1.19) | 0.8382 |
| *Diagnostic Lumbar Puncture* | 2.9 | 3.2 | 0.98  (0.54-1.81) | 0.9603 | 4.5 | 1.28  (0.95-1.71) | 0.105 | 6.4 | 1.85  (0.93-3.68) | 0.0774 | 2.4 | 0.81  (0.69-0.96) | 0.0137 |
| *Diagnostic Imaging* | 2.3 | 4.7 | 1.73  (1.03-2.92) | 0.0389 | 4.4 | 1.11  (0.82-1.50) | 0.4892 | 4.3 | 1.13  (0.49-2.61) | 0.7674 | 1.8 | 0.71  (0.59-0.86) | 0.0005 |
| *Digestive System Procedures* | 1.5 | 3.5 | 1.96  (1.09-3.54) | 0.025 | 3.0 | 1.71  (1.19-2.47) | 0.0039 | 5.0 | 2.79  (1.29-6.05) | 0.0094 | 1.5 | 1.02  (0.82-1.26) | 0.869 |
| *ENT and Eye Procedures* | 2.4 | 3.2 | 1.44  (0.78-2.66) | 0.2399 | 3.0 | 1.10  (0.77-1.57) | 0.6008 | 1.4 | 0.58  (0.14-2.34) | 0.4417 | 2.5 | 1.03  (0.87-1.22) | 0.7379 |
| *Respiratory System Procedures* | 1.1 | 1.7 | 1.41  (0.62-3.21) | 0.4137 | 2.0 | 1.53  (0.98-2.38) | 0.0625 | 2.9 | 2.22  (0.81-6.09) | 0.1223 | 1.0 | 0.93  (0.71-1.20) | 0.5532 |
| *Blood Transfusion* | .3 | .3 | 1.00  (0.14-7.26) | 0.9989 | 1.7 | 4.57  (2.67-7.83) | <.0001 | 2.1 | 6.38  (1.94-20.93) | 0.0022 | .5 | 1.87  (1.25-2.79) | 0.0023 |
| *Skin Procedures* | 1.5 | 2.6 | 1.52  (0.77-2.99) | 0.2244 | 2.7 | 1.13  (0.78-1.65) | 0.5138 | 2.9 | 1.27  (0.46-3.47) | 0.6418 | 1.9 | 1.14  (0.93-1.39) | 0.1979 |
| *Cardiovascular Procedures* | .7 | .6 | 0.87  (0.21-3.54) | 0.8457 | .9 | 1.45  (0.77-2.74) | 0.251 | .7 | 1.10  (0.15-7.99) | 0.9221 | .7 | 1.02  (0.74-1.40) | 0.9139 |
| *Musculoskeletal System Procedures* | .5 | .9 | 1.47  (0.46-4.69) | 0.5107 | .9 | 1.34  (0.71-2.53) | 0.3714 | 1.4 | 2.12  (0.51-8.73) | 0.2996 | .5 | 0.90  (0.62-1.29) | 0.5569 |
| *Nervous System Procedures* | .3 | .3 | 0.91  (0.13-6.60) | 0.925 | .3 | 0.80  (0.25-2.61) | 0.7135 | .0 | 0.00  (NA) | 0.8615 | .3 | 1.18  (0.75-1.85) | 0.4866 |
| *Electrocardiogram* | .4 | .9 | 2.03  (0.63-6.50) | 0.2351 | .9 | 1.24  (0.64-2.42) | 0.5237 | .7 | 1.20  (0.17-8.77) | 0.8549 | .6 | 1.31  (0.91-1.88) | 0.1452 |

**eTable 2:** Adjusted Odds Ratios for Hospital Admissions, Non-Routine Procedures, Emergency Department Visits, and Outpatient Clinic Services by Demographic Factors

|  | **Male** | | **Black or African American** | | **White** | | **Asian** | | **Hispanic or Latino** | | **Medicaid** | |
| --- | --- | --- | --- | --- | --- | --- | --- | --- | --- | --- | --- | --- |
|  | **AOR**  **(95% CI)** | **p** | **AOR**  **(95% CI)** | **p** | **AOR**  **(95% CI)** | **p** | **AOR**  **(95% CI)** | **p** | **AOR**  **(95% CI)** | **p** | **AOR**  **(95% CI)** | **p** |
| *Hospital Admissions* | 1.13  (1.04-1.23) | 0.0034 | 1.19  (1.04-1.36) | 0.0096 | 0.82  (0.72-0.94) | 0.0045 | 0.80  (0.63-1.01) | 0.0606 | 1.21  (1.04-1.40) | 0.0133 | 1.76  (1.45-2.14) | <.0001 |
| *Non-Routine Procedures* | 1.08  (1.01-1.15) | 0.0237 | 1.40  (1.26-1.55) | <.0001 | 0.84  (0.75-0.93) | 0.0011 | 0.65  (0.54-0.79) | <.0001 | 1.21  (1.07-1.36) | 0.0023 | 1.62  (1.40-1.87) | <.0001 |
| *Emergency Department Visits* | 1.13  (1.07-1.19) | <.0001 | 1.91  (1.76-2.08) | <.0001 | 0.58  (0.53-0.63) | <.0001 | 0.50  (0.42-0.58) | <.0001 | 2.03  (1.84-2.23) | <.0001 | 1.87  (1.64-2.14) | <.0001 |
| **Outpatient Clinic Services** | | | | | | | | | | | | |
| *Medical Specialty* | 1.04  (0.97-1.11) | 0.2295 | 1.18  (1.05-1.32) | 0.004 | 0.90  (0.80-1.00) | 0.0483 | 0.85  (0.71-1.03) | 0.096 | 1.12  (0.98-1.27) | 0.0893 | 1.53  (1.31-1.78) | <.0001 |
| *Surgical Specialty* | 2.41  (2.23-2.60) | <.0001 | 1.30  (1.15-1.47) | <.0001 | 1.12  (0.99-1.26) | 0.0679 | 0.70  (0.57-0.88) | 0.0016 | 0.93  (0.81-1.08) | 0.3301 | 1.69  (1.42-1.99) | <.0001 |
| *Neonatology* | 1.60  (1.43-1.79) | <.0001 | 2.18  (1.81-2.63) | <.0001 | 0.86  (0.71-1.05) | 0.1443 | 0.69  (0.48-0.99) | 0.0464 | 0.82  (0.65-1.05) | 0.1101 | 2.10  (1.67-2.65) | <.0001 |
| *Speech and Hearing* | 1.05  (0.94-1.17) | 0.385 | 1.16  (0.97-1.38) | 0.1072 | 0.89  (0.75-1.06) | 0.1928 | 0.93  (0.69-1.25) | 0.632 | 1.69  (1.41-2.04) | <.0001 | 1.15  (0.89-1.50) | 0.2853 |
| *Ophthalmology* | 0.88  (0.78-0.98) | 0.0263 | 0.92  (0.75-1.11) | 0.3735 | 1.00  (0.84-1.21) | 0.9595 | 1.01  (0.75-1.36) | 0.958 | 1.06  (0.85-1.31) | 0.606 | 1.57  (1.21-2.03) | 0.0006 |
| *Physical Therapy* | 0.97  (0.84-1.12) | 0.6799 | 1.24  (0.97-1.57) | 0.0825 | 1.32  (1.04-1.67) | 0.0208 | 1.75  (1.19-2.57) | 0.0044 | 1.31  (1.01-1.70) | 0.0395 | 79.86  (35.18-181.30) | <.0001 |
| *Labs and Imaging* | 0.89  (0.81-0.97) | 0.0091 | 0.99  (0.85-1.15) | 0.8951 | 1.37  (1.19-1.58) | <.0001 | 1.16  (0.89-1.50) | 0.2667 | 1.20  (1.02-1.42) | 0.0292 | 38.58  (22.93-64.92) | <.0001 |
| *Urgent Care* | 1.05  (0.96-1.14) | 0.2868 | 1.23  (1.08-1.41) | 0.0026 | 0.74  (0.64-0.84) | <.0001 | 0.92  (0.72-1.18) | 0.5208 | 1.54  (1.33-1.79) | <.0001 | 84.13  (59.10-119.77) | <.0001 |
| *Child Abuse and Neglect* | 1.07  (0.68-1.66) | 0.778 | 1.64  (0.82-3.29) | 0.1611 | 1.05  (0.48-2.29) | 0.9067 | 0.00  (0.00-inf) | 0.9998 | 0.70  (0.29-1.69) | 0.4313 | NA | 0.9996 |
| **Non-Routine Procedures** | | | | | | | | | | | | |
| *Echocardiogram* | 1.04  (0.93-1.17) | 0.462 | 1.65  (1.35-2.01) | <.0001 | 0.94  (0.77-1.15) | 0.5759 | 0.87  (0.61-1.23) | 0.4258 | 1.24  (0.99-1.55) | 0.0594 | .80 (.71-.91) | .0003 |
| *Enteral and Parenteral Nutrition* | 0.97  (0.85-1.10) | 0.6407 | 1.45  (1.17-1.80) | 0.0006 | 0.82  (0.66-1.01) | 0.0672 | 0.56  (0.36-0.86) | 0.0077 | 1.05  (0.82-1.35) | 0.7118 | .56 (.48-.64) | <.0001 |
| *Intubation and Ventilation* | 0.99  (0.87-1.13) | 0.9292 | 1.51  (1.21-1.87) | 0.0002 | 0.90  (0.72-1.12) | 0.3289 | 0.89  (0.61-1.30) | 0.5519 | 1.00  (0.78-1.30) | 0.9698 | 1.63  (1.26-2.10) | 0.0002 |
| *Vascular Catheterization (not heart)* | 0.95  (0.83-1.09) | 0.4863 | 1.66  (1.31-2.10) | <.0001 | 0.88  (0.69-1.12) | 0.2936 | 0.69  (0.44-1.08) | 0.1049 | 1.08  (0.82-1.43) | 0.5755 | 1.34  (1.00-1.80) | 0.0482 |
| *Diagnostic Lumbar Puncture* | 1.27  (1.10-1.46) | 0.0009 | 0.93  (0.75-1.16) | 0.5341 | 0.66  (0.53-0.82) | 0.0002 | 0.53  (0.35-0.82) | 0.0038 | 1.20  (0.95-1.53) | 0.1309 | 1.79  (1.33-2.41) | 0.0001 |
| *Diagnostic Imaging* | 1.37  (1.17-1.60) | 0.0001 | 1.90  (1.45-2.50) | <.0001 | 0.72  (0.54-0.96) | 0.0228 | 0.53  (0.31-0.92) | 0.0231 | 1.44  (1.06-1.95) | 0.0193 | 1.62  (1.20-2.18) | 0.0016 |
| *Digestive System Procedures* | 1.41  (1.18-1.70) | 0.0002 | 1.25  (0.93-1.69) | 0.143 | 0.90  (0.67-1.22) | 0.4983 | 0.70  (0.39-1.25) | 0.2269 | 1.24  (0.89-1.72) | 0.2102 | 1.31  (0.94-1.83) | 0.1055 |
| *ENT and Eye Procedures* | 0.69  (0.60-0.80) | <.0001 | 1.31  (1.02-1.69) | 0.0371 | 1.04  (0.81-1.33) | 0.7609 | 0.74  (0.47-1.16) | 0.1867 | 1.50  (1.14-1.97) | 0.0038 | 2.16  (1.61-2.89) | <.0001 |
| *Respiratory System Procedures* | 1.15  (0.92-1.43) | 0.2173 | 1.26  (0.88-1.81) | 0.2059 | 0.91  (0.64-1.31) | 0.6129 | 0.26  (0.09-0.74) | 0.0111 | 1.30  (0.88-1.94) | 0.1887 | 2.35  (1.61-3.43) | <.0001 |
| *Blood Transfusion* | 0.97  (0.67-1.40) | 0.8691 | 1.69  (0.90-3.18) | 0.101 | 0.79  (0.41-1.53) | 0.4887 | 0.60  (0.17-2.15) | 0.4331 | 0.88  (0.40-1.92) | 0.7422 | 1.10  (0.78-1.56) | 0.575 |
| *Skin Procedures* | 1.22  (1.02-1.46) | 0.0289 | 1.52  (1.11-2.08) | 0.0085 | 0.90  (0.66-1.24) | 0.5266 | 0.78  (0.46-1.34) | 0.3724 | 1.07  (0.74-1.53) | 0.7247 | 2.95  (1.96-4.44) | <.0001 |
| *Cardiovascular Procedures* | 0.91  (0.69-1.20) | 0.5083 | 1.56  (0.96-2.53) | 0.0736 | 0.96  (0.59-1.57) | 0.8857 | 1.16  (0.53-2.56) | 0.71 | 1.55  (0.92-2.61) | 0.1016 | 1.01  (0.39-2.58) | 0.9884 |
| *Musculoskeletal System Procedures* | 1.13  (0.83-1.54) | 0.4518 | 1.24  (0.73-2.10) | 0.4215 | 1.07  (0.64-1.80) | 0.7941 | 0.26  (0.06-1.13) | 0.0733 | 0.92  (0.50-1.71) | 0.7956 | 1.91  (1.34-2.73) | 0.0004 |
| *Nervous System Procedures* | 1.28  (0.84-1.95) | 0.2446 | 1.62  (0.78-3.34) | 0.1942 | 1.16  (0.57-2.34) | 0.6834 | 0.27  (0.03-2.16) | 0.2182 | 1.84  (0.86-3.91) | 0.1159 | 1.00  (0.46-2.15) | 0.9942 |
| *Electrocardiogram* | 0.92  (0.66-1.28) | 0.6166 | 1.23  (0.70-2.16) | 0.4657 | 0.69  (0.39-1.22) | 0.2053 | 0.68  (0.26-1.75) | 0.4252 | 0.97  (0.50-1.89) | 0.9283 | 2.53  (1.40-4.60) | 0.0022 |

**eTable 3:** Adjusted Odds Ratios and Rates of Outpatient Specialty Care by Diagnosis

|  | **No Dx** | **ASD** | | | **ADHD** | | | **ASD+ADHD** | | | **URI** | | |
| --- | --- | --- | --- | --- | --- | --- | --- | --- | --- | --- | --- | --- | --- |
|  | **%** | **%** | **AOR**  **(95% CI)** | **p** | **%** | **AOR**  **(95% CI)** | **p** | **%** | **AOR**  **(95% CI)** | **p** | **%** | **AOR**  **(95% CI)** | **p** |
| **Inpatient Services** | | | | | | | | | | | | | |
| *Pediatrics* | 7.1 | 11.4 | 1.51  (1.08-2.13) | 0.0171 | 11 | 1.57  (1.29-1.91) | <.0001 | 9.3 | 1.25  (0.70-2.23) | 0.4453 | 5.9 | 0.83  (0.74-0.93) | 0.0008 |
| *Neonatology* | 1 | 0.9 | 0.81  (0.26-2.56) | 0.7239 | 2 | 2.06  (1.32-3.21) | 0.0014 | 2.1 | 2.12  (0.67-6.77) | 0.2025 | 0.9 | 0.93  (0.71-1.22) | 0.6036 |
| *Medical Specialty* | 0.7 | 0.6 | 0.80  (0.20-3.27) | 0.761 | 1 | 1.23  (0.67-2.25) | 0.5049 | 1.4 | 1.76  (0.43-7.22) | 0.4329 | 0.7 | 1.04  (0.77-1.42) | 0.7962 |
| *Surgical Specialty* | 0.8 | 0.9 | 1.11  (0.35-3.51) | 0.8585 | 0.9 | 1.22  (0.63-2.36) | 0.5544 | 0.7 | 0.99  (0.14-7.14) | 0.9896 | 0.7 | 0.87  (0.64-1.19) | 0.3926 |
| **Medical Specialty Care** | | | | | | | | | | | | | |
| *Cardiology* | 4.4 | 5.2 | 1.16  (0.72-1.88) | 0.5421 | 7.1 | 1.53  (1.21-1.95) | 0.0004 | 5.7 | 1.23  (0.60-2.53) | 0.5702 | 4.6 | 1.04  (0.92-1.18) | 0.54 |
| *Medicine* | 3.4 | 7.3 | 2.09  (1.36-3.20) | 0.0007 | 8.3 | 1.47  (1.17-1.84) | 0.0008 | 5 | 0.96  (0.44-2.07) | 0.91 | 2.9 | 0.79  (0.68-0.92) | 0.0026 |
| *Dermatology* | 2.2 | 3.8 | 1.70  (0.97-2.99) | 0.0653 | 2.6 | 1.17  (0.80-1.72) | 0.413 | 2.1 | 0.98  (0.31-3.10) | 0.9746 | 2.3 | 1.02  (0.86-1.22) | 0.8245 |
| *Allergy and Pulmonology* | 1.8 | 2 | 1.03  (0.48-2.20) | 0.9407 | 2.5 | 1.32  (0.89-1.95) | 0.1711 | 5 | 2.59  (1.20-5.61) | 0.0156 | 1.9 | 1.03  (0.85-1.25) | 0.7391 |
| *Neurology* | 1.2 | 3.8 | 3.15  (1.77-5.58) | <.0001 | 2.1 | 1.90  (1.24-2.93) | 0.0034 | 3.6 | 3.21  (1.29-7.97) | 0.0119 | 1.1 | 0.88  (0.68-1.13) | 0.3147 |
| *Gastroenterology* | 1.1 | 3.5 | 3.25  (1.79-5.91) | 0.0001 | 1.3 | 1.42  (0.82-2.44) | 0.2065 | 1.4 | 1.45  (0.36-5.95) | 0.6019 | 1 | 0.95  (0.73-1.23) | 0.6963 |
| *Endocrinology* | 1.1 | 3.8 | 3.39  (1.91-6.03) | <.0001 | 2 | 1.54  (0.99-2.40) | 0.0578 | 3.6 | 2.93  (1.18-7.27) | 0.0206 | 1.3 | 1.22  (0.97-1.55) | 0.0916 |
| *Heme/Onc* | 0.6 | 0.6 | 0.82  (0.20-3.33) | 0.7803 | 0.9 | 1.20  (0.64-2.26) | 0.5689 | 2.9 | 3.73  (1.35-10.33) | 0.0113 | 0.8 | 1.21  (0.89-1.64) | 0.2292 |
| **Surgical Specialty Care** | | | | | | | | | | | | | |
| *General Surgery* | 3.6 | 7 | 1.61  (1.05-2.47) | 0.0301 | 4.1 | 1.73  (1.27-2.37) | 0.0006 | 2.9 | 0.84  (0.31-2.31) | 0.7406 | 3.2 | 0.98  (0.84-1.13) | 0.7681 |
| *Ear, Nose, and Throat* | 2.7 | 5.8 | 2.01  (1.26-3.19) | 0.0031 | 2.6 | 1.16  (0.79-1.70) | 0.4536 | 3.6 | 1.38  (0.56-3.40) | 0.4824 | 2.7 | 1.02  (0.86-1.19) | 0.8436 |
| *Orthopaedics* | 1.7 | 2.3 | 1.47  (0.72-2.99) | 0.291 | 2.1 | 1.35  (0.89-2.06) | 0.1612 | 2.1 | 1.42  (0.45-4.49) | 0.5521 | 1.8 | 1.06  (0.87-1.29) | 0.5932 |
| *Plastic Surgery* | 1.2 | 2.6 | 2.06  (1.04-4.05) | 0.037 | 1.4 | 1.25  (0.74-2.11) | 0.4055 | 0.7 | 0.59  (0.08-4.26) | 0.6024 | 1.3 | 1.15  (0.91-1.45) | 0.2405 |
| *Neurosurgery* | 1 | 1.5 | 1.37  (0.56-3.37) | 0.4887 | 0.9 | 0.82  (0.43-1.58) | 0.5603 | 0.7 | 0.67  (0.09-4.83) | 0.6908 | 0.9 | 0.91  (0.69-1.19) | 0.4704 |
| **Vision, Speech, and Hearing Specialty Care** | | | | | | | | | | | | | |
| *Ophthalmology* | 3.8 | 12 | 3.45  (2.47-4.84) | <.0001 | 6.6 | 1.74  (1.35-2.23) | <.0001 | 9.3 | 2.61  (1.46-4.66) | 0.0012 | 4.3 | 1.11  (0.97-1.26) | 0.1224 |
| *Speech Pathology* | 3.2 | 7.9 | 2.52  (1.67-3.79) | <.0001 | 6.7 | 1.45  (1.13-1.85) | 0.0033 | 8.6 | 2.06  (1.12-3.76) | 0.0194 | 3.4 | 1.03  (0.89-1.19) | 0.7296 |
| *Audiology* | 1.6 | 2.6 | 1.52  (0.76-3.00) | 0.2334 | 0.6 | 2.36  (1.08-5.18) | 0.0323 | 0 | 0.00  (NA) | 0.9919 | 1.5 | 1.08  (0.88-1.34) | 0.4617 |

**eTable 4:** Adjusted Odds Ratios for Outpatient Specialty Care by Demographic Factors

|  | **Male** | | **Black or African American** | | **White** | | **Asian** | | **Hispanic or Latino** | | **Medicaid** | |
| --- | --- | --- | --- | --- | --- | --- | --- | --- | --- | --- | --- | --- |
|  | **AOR**  **(95% CI)** | **p** | **AOR**  **(95% CI)** | **p** | **AOR**  **(95% CI)** | **p** | **AOR**  **(95% CI)** | **p** | **AOR**  **(95% CI)** | **p** | **AOR**  **(95% CI)** | **p** |
| **Inpatient Services** | | | | | | | | | | | | |
| *Pediatrics* | 1.14  (1.04 - 1.25) | 0.0053 | 1.11  (0.96 - 1.28) | 0.171 | 0.75  (0.65 - 0.87) | 0.0001 | 0.82  (0.64 - 1.06) | 0.1231 | 1.24  (1.05 - 1.46) | 0.0097 | 1.87  (1.52 - 2.31) | <.0001 |
| *Neonatology* | 1.09  (0.87 - 1.37) | 0.4609 | 1.44  (0.98 - 2.13) | 0.0645 | 1.10  (0.74 - 1.64) | 0.6224 | 0.62  (0.27 - 1.42) | 0.2581 | 0.86  (0.54 - 1.37) | 0.5194 | 1.59  (0.87 - 2.87) | 0.1289 |
| *Medical Specialty* | 1.10  (0.84 - 1.44) | 0.4925 | 1.91  (1.15 - 3.18) | 0.0124 | 1.43  (0.87 - 2.34) | 0.1616 | 1.55  (0.73 - 3.30) | 0.2521 | 1.57  (0.92 - 2.70) | 0.1012 | 0.88  (0.43 - 1.78) | 0.7156 |
| *Surgical Specialty* | 0.94  (0.72 - 1.22) | 0.631 | 1.43  (0.91 - 2.26) | 0.1212 | 1.21  (0.77 - 1.89) | 0.4012 | 0.48  (0.16 - 1.38) | 0.1715 | 1.39  (0.85 - 2.27) | 0.1907 | 0.95  (0.43 - 2.09) | 0.8934 |
| **Medical Specialty Care** | | | | | | | | | | | | |
| *Cardiology* | 0.94  (0.84-1.05) | 0.2485 | 1.37  (1.13-1.65) | 0.0011 | 0.99  (0.83-1.20) | 0.9473 | 0.88  (0.63-1.21) | 0.4227 | 1.38  (1.12-1.70) | 0.0021 | 1.46  (1.13-1.89) | 0.0034 |
| *Medicine* | 1.10  (0.97-1.25) | 0.1472 | 0.91  (0.74-1.11) | 0.3498 | 0.64  (0.52-0.78) | <.0001 | 0.42  (0.28-0.63) | <.0001 | 1.09  (0.86-1.37) | 0.4834 | 1.56  (1.18-2.07) | 0.0018 |
| *Dermatology* | 0.94  (0.81-1.10) | 0.4488 | 0.99  (0.77-1.27) | 0.9063 | 0.75  (0.59-0.97) | 0.0256 | 1.27  (0.89-1.83) | 0.1893 | 0.98  (0.73-1.32) | 0.9081 | 1.59  (1.13-2.23) | 0.0079 |
| *Allergy and Pulmonology* | 1.40  (1.18-1.67) | 0.0001 | 0.79  (0.59-1.06) | 0.1205 | 1.06  (0.81-1.38) | 0.6578 | 1.03  (0.67-1.58) | 0.8904 | 0.83  (0.59-1.17) | 0.2918 | 1.67  (1.16-2.41) | 0.006 |
| *Neurology* | 0.87  (0.71-1.08) | 0.2027 | 1.16  (0.82-1.64) | 0.3967 | 0.99  (0.70-1.39) | 0.9522 | 0.85  (0.46-1.57) | 0.6141 | 1.11  (0.75-1.64) | 0.5997 | 2.61  (1.72-3.97) | <.0001 |
| *Gastroenterology* | 1.10  (0.88-1.37) | 0.4074 | 1.40  (0.92-2.11) | 0.1149 | 1.83  (1.24-2.70) | 0.0023 | 1.17  (0.59-2.30) | 0.657 | 1.28  (0.82-1.99) | 0.2708 | 2.67  (1.68-4.25) | <.0001 |
| *Endocrinology* | 1.02  (0.82-1.25) | 0.8888 | 1.14  (0.81-1.62) | 0.4541 | 0.92  (0.65-1.30) | 0.6421 | 0.56  (0.28-1.11) | 0.0969 | 1.25  (0.85-1.84) | 0.251 | 2.18  (1.44-3.29) | 0.0002 |
| *Heme/Onc* | 1.28  (0.97-1.69) | 0.0863 | 1.60  (0.99-2.58) | 0.0566 | 0.90  (0.55-1.47) | 0.6737 | 1.22  (0.58-2.55) | 0.5985 | 1.05  (0.60-1.86) | 0.8624 | 1.58  (0.87-2.86) | 0.1347 |
| **Surgical Specialty Care** | | | | | | | | | | | | |
| *General Surgery* | 2.60  (2.26-2.99) | <.0001 | 1.52  (1.23-1.87) | <.0001 | 1.04  (0.85-1.29) | 0.6923 | 0.74  (0.49-1.12) | 0.1529 | 0.83  (0.64-1.08) | 0.1677 | 2.34  (1.67-3.29) | <.0001 |
| *Ear, Nose, and Throat* | 1.50  (1.30-1.73) | <.0001 | 1.02  (0.80-1.30) | 0.8581 | 1.15  (0.92-1.44) | 0.2192 | 0.63  (0.40-0.99) | 0.0434 | 1.31  (1.01-1.70) | 0.0413 | 1.82  (1.32-2.50) | 0.0002 |
| *Orthopaedics* | 0.76  (0.64-0.91) | 0.0022 | 0.97  (0.72-1.31) | 0.8482 | 1.17  (0.89-1.55) | 0.2657 | 0.56  (0.31-1.01) | 0.0547 | 0.91  (0.65-1.28) | 0.598 | 1.93  (1.31-2.84) | 0.0008 |
| *Plastic Surgery* | 1.34  (1.09-1.66) | 0.0058 | 1.16  (0.82-1.63) | 0.4042 | 0.99  (0.71-1.38) | 0.9491 | 0.72  (0.39-1.35) | 0.3114 | 0.87  (0.58-1.32) | 0.5196 | 1.36  (0.81-2.29) | 0.2511 |
| *Neurosurgery* | 1.21  (0.96-1.52) | 0.1145 | 0.68  (0.46-1.01) | 0.0547 | 1.11  (0.78-1.58) | 0.5618 | 0.67  (0.34-1.32) | 0.2517 | 0.95  (0.62-1.46) | 0.8143 | 1.71  (1.02-2.88) | 0.0436 |
| **Vision, Speech, and Hearing Specialty Care** | | | | | | | | | | | | |
| *Ophthalmology* | 0.88  (0.78-0.98) | 0.0263 | 0.92  (0.75-1.11) | 0.3735 | 1.00  (0.84-1.21) | 0.9595 | 1.01  (0.75-1.36) | 0.958 | 1.06  (0.85-1.31) | 0.606 | 1.57  (1.21-2.03) | 0.0006 |
| *Speech Pathology* | 1.03  (0.90-1.16) | 0.6962 | 1.12  (0.90-1.39) | 0.3078 | 0.84  (0.68-1.04) | 0.1127 | 0.89  (0.63-1.26) | 0.5213 | 1.75  (1.40-2.19) | <.0001 | 1.05  (0.78-1.41) | 0.7708 |
| *Audiology* | 1.08  (0.90-1.30) | 0.4102 | 1.28  (0.95-1.73) | 0.1104 | 1.15  (0.85-1.55) | 0.3719 | 1.15  (0.66-2.00) | 0.6212 | 1.74  (1.27-2.40) | 0.0006 | 33.19  (8.61-128.04) | <.0001 |

**eTable 5:** Adjusted Odds Ratios and Rates of Procedures from Birth and Non-Birth Encounters by Diagnosis

|  | **No Dx** | **ASD** | | | **ADHD** | | | **ASD+ADHD** | | | **URI** | | |
| --- | --- | --- | --- | --- | --- | --- | --- | --- | --- | --- | --- | --- | --- |
|  | **%** | **%** | **AOR**  **(95% CI)** | **p** | **%** | **AOR**  **(95% CI)** | **p** | **%** | **AOR**  **(95% CI)** | **p** | **%** | **AOR**  **(95% CI)** | **p** |
| **Procedures from Birth Encounters** | | | | | | | | | | | | | |
| *Echocardiogram* | 2.7 | 5 | 1.76  (1.07-2.89) | 0.0262 | 4.7 | 1.77  (1.32-2.37) | 0.0001 | 5.7 | 2.14  (1.04-4.41) | 0.0395 | 2.6 | 0.95  (0.80-1.11) | 0.5002 |
| *Enteral and Parenteral Nutrition* | 2.6 | 6.1 | 2.35  (1.49-3.71) | 0.0002 | 6.5 | 2.01  (1.55-2.59) | <.0001 | 5.7 | 1.88  (0.91-3.88) | 0.0867 | 2.9 | 1.10  (0.94-1.29) | 0.251 |
| *Intubation and Ventilation* | 2.9 | 7.6 | 2.56  (1.70-3.86) | <.0001 | 4.4 | 1.56  (1.16-2.10) | 0.0037 | 5 | 1.73  (0.80-3.72) | 0.1644 | 2.7 | 0.93  (0.79-1.09) | 0.3428 |
| *Vascular Catheterization (not heart)* | 2.3 | 5.2 | 2.22  (1.36-3.61) | 0.0014 | 4.3 | 1.59  (1.17-2.16) | 0.003 | 5.7 | 2.27  (1.10-4.68) | 0.0273 | 2.3 | 0.99  (0.84-1.18) | 0.9381 |
| *Diagnostic Lumbar Puncture* | 1.1 | 0.6 | 0.47  (0.12-1.90) | 0.2876 | 1.4 | 0.99  (0.59-1.67) | 0.9825 | 2.9 | 2.11  (0.77-5.77) | 0.1481 | 1.1 | 1.01  (0.79-1.30) | 0.9299 |
| *Diagnostic Imaging* | 0.3 | 0.9 | 2.87  (0.89-9.27) | 0.0788 | 0.8 | 2.21  (1.06-4.60) | 0.0336 | 0.7 | 1.98  (0.27-14.54) | 0.5015 | 0.3 | 1.27  (0.79-2.03) | 0.3259 |
| *Digestive System Procedures* | 0.6 | 2.6 | 3.88  (1.94-7.76) | 0.0001 | 1 | 1.73  (0.94-3.21) | 0.0789 | 1.4 | 2.26  (0.55-9.31) | 0.2591 | 0.6 | 0.99  (0.71-1.40) | 0.9658 |
| *ENT and Eye Procedures* | 0.7 | 1.2 | 1.71  (0.63-4.69) | 0.2934 | 0.3 | 0.98  (0.36-2.71) | 0.9703 | 0.7 | 1.56  (0.22-11.37) | 0.6582 | 0.8 | 1.33  (0.99-1.78) | 0.0592 |
| *Respiratory System Procedures* | 0.4 | 0.9 | 1.83  (0.57-5.84) | 0.31 | 0.7 | 1.80  (0.85-3.83) | 0.1234 | 1.4 | 3.50  (0.84-14.55) | 0.0847 | 0.5 | 1.17  (0.79-1.73) | 0.4297 |
| *Blood Transfusion* | 0.1 | 0 | 0.00  (NA) | 0.9961 | 0.9 | 7.32  (3.38-15.88) | <.0001 | 0.7 | 6.00  (0.79-45.70) | 0.0838 | 0.2 | 2.06  (1.11-3.82) | 0.0223 |
| **Procedures from Non-Birth Encounters** | | | | | | | | | | | | | |
| *Echocardiogram* | 1.7 | 2.6 | 1.40  (0.71-2.75) | 0.327 | 3.1 | 1.28  (0.90-1.83) | 0.1681 | 5 | 2.26  (1.04-4.89) | 0.0393 | 2 | 1.11  (0.92-1.34) | 0.2829 |
| *Enteral and Parenteral Nutrition* | 0.8 | 1.7 | 2.29  (1.00-5.23) | 0.0503 | 2.2 | 2.19  (1.42-3.38) | 0.0004 | 3.6 | 4.08  (1.63-10.22) | 0.0026 | 0.7 | 0.86  (0.63-1.17) | 0.334 |
| *Intubation and Ventilation* | 0.6 | 0.3 | 0.51  (0.07-3.65) | 0.5001 | 1.2 | 1.89  (1.06-3.38) | 0.0308 | 0.7 | 1.17  (0.16-8.53) | 0.8737 | 0.3 | 0.52  (0.33-0.81) | 0.0039 |
| *Vascular Catheterization (not heart)* | 0.6 | 0 | 0.00  (NA) | 0.6223 | 1.6 | 2.23  (1.34-3.71) | 0.0019 | 1.4 | 2.09  (0.51-8.60) | 0.3087 | 0.5 | 0.96  (0.67-1.36) | 0.8028 |
| *Diagnostic Lumbar Puncture* | 1.9 | 2.6 | 1.27  (0.65-2.49) | 0.4868 | 3.2 | 1.43  (1.01-2.02) | 0.0459 | 3.6 | 1.59  (0.64-3.92) | 0.3158 | 1.3 | 0.68  (0.55-0.85) | 0.0007 |
| *Diagnostic Imaging* | 2.1 | 3.8 | 1.55  (0.87-2.76) | 0.1339 | 3.7 | 0.99  (0.72-1.38) | 0.9726 | 3.6 | 1.03  (0.42-2.57) | 0.9427 | 1.5 | 0.66  (0.53-0.81) | <.0001 |
| *Digestive System Procedures* | 1.1 | 2 | 1.62  (0.76-3.48) | 0.2142 | 2.2 | 1.72  (1.13-2.63) | 0.012 | 4.3 | 3.28  (1.42-7.56) | 0.0053 | 1.1 | 1.07  (0.83-1.37) | 0.5958 |
| *ENT and Eye Procedures* | 1.7 | 2 | 1.30  (0.61-2.78) | 0.5029 | 2.6 | 1.11  (0.76-1.61) | 0.602 | 0.7 | 0.35  (0.05-2.55) | 0.3028 | 1.7 | 0.92  (0.75-1.13) | 0.4301 |
| *Respiratory System Procedures* | 0.7 | 0.9 | 1.04  (0.33-3.29) | 0.9489 | 1.4 | 1.38  (0.81-2.35) | 0.2371 | 1.4 | 1.47  (0.36-6.03) | 0.5949 | 0.6 | 0.84  (0.61-1.16) | 0.2897 |
| *Blood Transfusion* | 0.2 | 0.3 | 1.57  (0.21-11.56) | 0.6575 | 0.8 | 2.88  (1.35-6.13) | 0.006 | 1.4 | 6.16  (1.44-26.30) | 0.0142 | 0.4 | 1.90  (1.16-3.13) | 0.0109 |

**eTable 6:** Adjusted Odds Ratios for Procedures from Birth and Non-Birth Encounters by Demographic Factors

|  | **Male** | | **Black or African American** | | **White** | | **Asian** | | **Hispanic or Latino** | | **Medicaid** | |
| --- | --- | --- | --- | --- | --- | --- | --- | --- | --- | --- | --- | --- |
|  | **AOR**  **(95% CI)** | **p** | **AOR**  **(95% CI)** | **p** | **AOR**  **(95% CI)** | **p** | **AOR**  **(95% CI)** | **p** | **AOR**  **(95% CI)** | **p** | **AOR**  **(95% CI)** | **p** |
| **Procedures from Birth Encounters** | | | | | | | | | | | | |
| *Echocardiogram* | 0.96  (0.84-1.11) | 0.5932 | 1.76  (1.38-2.23) | <.0001 | 0.94  (0.74-1.21) | 0.649 | 0.89  (0.58-1.37) | 0.6057 | 1.28  (0.97-1.68) | 0.0782 | 1.63  (1.18-2.24) | 0.0029 |
| *Enteral and Parenteral Nutrition* | 1.00  (0.87-1.14) | 0.9649 | 1.39  (1.10-1.75) | 0.0052 | 0.78  (0.61-0.98) | 0.0336 | 0.56  (0.36-0.89) | 0.0134 | 1.00  (0.76-1.32) | 0.9751 | 1.29  (0.94-1.77) | 0.1194 |
| *Intubation and Ventilation* | 1.03  (0.90-1.18) | 0.6515 | 1.45  (1.16-1.81) | 0.0013 | 0.87  (0.69-1.09) | 0.2256 | 0.86  (0.57-1.28) | 0.4509 | 0.98  (0.75-1.28) | 0.8687 | 1.80  (1.32-2.46) | 0.0002 |
| *Vascular Catheterization (not heart)* | 0.93  (0.80-1.08) | 0.3664 | 1.74  (1.35-2.25) | <.0001 | 0.87  (0.67-1.13) | 0.3078 | 0.64  (0.39-1.06) | 0.0834 | 1.11  (0.82-1.50) | 0.4947 | 1.73  (1.26-2.37) | 0.0007 |
| *Diagnostic Lumbar Puncture* | 1.36  (1.09-1.71) | 0.0065 | 0.92  (0.65-1.31) | 0.6487 | 0.68  (0.48-0.96) | 0.0272 | 0.55  (0.28-1.06) | 0.0758 | 1.05  (0.71-1.55) | 0.824 | 1.59  (0.97-2.59) | 0.0633 |
| *Diagnostic Imaging* | 1.94  (1.24-3.03) | 0.0035 | 0.65  (0.33-1.28) | 0.2106 | 0.86  (0.46-1.60) | 0.6293 | 0.70  (0.23-2.17) | 0.5343 | 0.90  (0.42-1.92) | 0.7907 | 0.98  (0.33-2.90) | 0.9758 |
| *Digestive System Procedures* | 1.10  (0.83-1.47) | 0.5056 | 1.54  (0.94-2.53) | 0.088 | 1.22  (0.75-1.99) | 0.4304 | 0.67  (0.23-1.97) | 0.4658 | 2.00  (1.21-3.30) | 0.0067 | 3.24  (1.83-5.72) | <.0001 |
| *ENT and Eye Procedures* | 0.92  (0.70-1.20) | 0.5213 | 1.70  (1.07-2.72) | 0.0255 | 1.17  (0.74-1.86) | 0.5079 | 1.49  (0.72-3.08) | 0.2873 | 1.60  (0.96-2.66) | 0.0725 | 0.98  (0.39-2.48) | 0.9621 |
| *Respiratory System Procedures* | 1.21  (0.85-1.71) | 0.2871 | 1.03  (0.58-1.84) | 0.911 | 1.38  (0.79-2.43) | 0.2559 | 0.46  (0.10-2.01) | 0.2993 | 1.08  (0.58-2.02) | 0.8004 | 4.50  (2.22-9.13) | <.0001 |
| *Blood Transfusion* | 0.91  (0.52-1.58) | 0.7296 | 2.63  (0.90-7.64) | 0.0764 | 1.18  (0.39-3.54) | 0.7662 | 0.79  (0.09-7.17) | 0.8314 | 1.55  (0.48-4.99) | 0.4658 | 0.21  (0.02-2.22) | 0.1942 |
| **Procedures from Non-Birth Encounters** | | | | | | | | | | | | |
| *Echocardiogram* | 1.11  (0.94-1.32) | 0.2244 | 1.44  (1.07-1.94) | 0.0147 | 0.93  (0.69-1.24) | 0.6112 | 0.78  (0.47-1.30) | 0.3422 | 1.34  (0.97-1.86) | 0.0748 | 1.74  (1.23-2.47) | 0.0019 |
| *Enteral and Parenteral Nutrition* | 0.77  (0.60-0.99) | 0.0418 | 1.91  (1.22-3.01) | 0.0049 | 1.05  (0.66-1.66) | 0.8332 | 0.33  (0.10-1.09) | 0.0699 | 1.29  (0.78-2.15) | 0.3238 | 1.27  (0.71-2.28) | 0.4259 |
| *Intubation and Ventilation* | 0.93  (0.68-1.29) | 0.6711 | 1.82  (1.03-3.24) | 0.04 | 1.07  (0.60-1.91) | 0.8209 | 0.91  (0.33-2.53) | 0.8543 | 1.35  (0.71-2.55) | 0.3645 | 1.14  (0.51-2.55) | 0.7456 |
| *Vascular Catheterization (not heart)* | 1.07  (0.79-1.44) | 0.6769 | 1.49  (0.89-2.50) | 0.1313 | 0.86  (0.51-1.45) | 0.5669 | 0.87  (0.36-2.09) | 0.7542 | 1.11  (0.61-2.03) | 0.7356 | 0.97  (0.47-2.04) | 0.9422 |
| *Diagnostic Lumbar Puncture* | 1.19  (1.00-1.41) | 0.0554 | 0.93  (0.71-1.23) | 0.6246 | 0.65  (0.49-0.85) | 0.0018 | 0.51  (0.29-0.88) | 0.0158 | 1.31  (0.97-1.75) | 0.0752 | 1.09  (0.70-1.69) | 0.6969 |
| *Diagnostic Imaging* | 1.28  (1.09-1.52) | 0.0034 | 2.20  (1.63-2.97) | <.0001 | 0.70  (0.51-0.96) | 0.0268 | 0.50  (0.27-0.93) | 0.0284 | 1.64  (1.18-2.28) | 0.0033 | 2.36  (1.75-3.20) | <.0001 |
| *Digestive System Procedures* | 1.51  (1.21-1.88) | 0.0002 | 1.18  (0.83-1.68) | 0.355 | 0.88  (0.62-1.26) | 0.4961 | 0.67  (0.34-1.31) | 0.2428 | 0.95  (0.63-1.43) | 0.7997 | 2.00  (1.25-3.19) | 0.0036 |
| *ENT and Eye Procedures* | 0.62  (0.52-0.74) | <.0001 | 1.16  (0.86-1.58) | 0.3281 | 0.98  (0.73-1.31) | 0.8982 | 0.51  (0.28-0.92) | 0.0257 | 1.43  (1.04-1.98) | 0.0283 | 1.17  (0.78-1.74) | 0.4526 |
| *Respiratory System Procedures* | 1.14  (0.87-1.49) | 0.3489 | 1.47  (0.93-2.31) | 0.0966 | 0.77  (0.49-1.22) | 0.2606 | 0.18  (0.04-0.78) | 0.0214 | 1.47  (0.90-2.42) | 0.1277 | 2.43  (1.49-3.95) | 0.0003 |
| *Blood Transfusion* | 0.97  (0.61-1.53) | 0.8851 | 1.38  (0.64-2.97) | 0.4061 | 0.67  (0.30-1.49) | 0.3291 | 0.73  (0.19-2.77) | 0.6465 | 0.56  (0.19-1.64) | 0.2911 | 1.28  (0.46-3.61) | 0.6354 |

**eTable 7:** Length of Hospital Stay after Birth and after Non-Birth Admissions by Diagnosis

|  | **No Dx** | **ASD** | **ADHD** | **ASD+ADHD** | **URI** |
| --- | --- | --- | --- | --- | --- |
| **Length of Stay after Birth** | | | | | |
| *median* | 2.28 | 2.55 | 2.44 | 2.52 | 2.28 |
| **Pairwise Comparisons**  (Mann-Whitney *U* Statistic; p-value) | | | | | |
| *ASD* | *U=*3.3x10^6^  *p<.0001* |  |  |  |  |
| *ADHD* | *U=*1.1x10^7^  *p<.0001* | *U=*1.6x10^5^  *p=*0.0215 |  |  |  |
| *ASD+ADHD* | *U=*1.2x10^6^  *p=*0.0310 | *U=*1.8x10^4^  *p=*0.3307 | *U=*6.7x10^4^  *p=*0.7362 |  |  |
| *URI* | *U=*6.3x10^7^  *p=*0.4244 | *U=*9.1x10^5^  *p<.0001* | *U=*3.5x10^6^  *p<.0001* | *U=*3.7x10^5^  *p=*0.0440 |  |
| **Length of Stay after Non-Birth Admission** | | | | | |
| *median* | 2.67 | 2.925 | 3.76 | 5.09 | 3.07 |
| **Pairwise Comparisons**  (Mann-Whitney *U* Statistic; p-value) | | | | | |
| *ASD* | *U=*6.8x10^4^  *p=*0.4057 |  |  |  |  |
| *ADHD* | *U=*2.7x10^5^  *p<.0001* | *U=*6.2x10^3^  *p=*0.2169 |  |  |  |
| *ASD+ADHD* | *U=*4.4x10^4^  *p=*0.0032 | *U=*1.0x10^3^  *p=*0.0881 | *U=*3.3x10^3^  *p=*0.2674 |  |  |
| *URI* | U=9.2x10^5^  *p=*0.0007 | *U=*2.1x10^4^  *p=*0.8542 | *U=*6.7x10^0^  *p=*0.0337 | *U=*8.3x10^3^  *p=*0.0368 |  |

**eTable 8:** Group Definitions for Outpatient Clinic Encounters

| **Inpatient Service** | **Service Group** |
| --- | --- |
| Emergency Medicine | ED Only |
| Not Necessary | ED Only |
| Pediatric Emergency Medicine | ED Only |
| Pediatric Cardiology | Medical Specialty |
| Ped Hematol-Oncology | Medical Specialty |
| Pediatric Neurology | Medical Specialty |
| Internal Medicine | Medical Specialty |
| Ped Gastroenterology | Medical Specialty |
| Pediatric Pulmonary | Medical Specialty |
| Pediatric Nephrology | Medical Specialty |
| Ped Endocrinology | Medical Specialty |
| Ped Infect. Diseases | Medical Specialty |
| Gastroenterology | Medical Specialty |
| Cardiology | Medical Specialty |
| Ophthalmology | Medical Specialty |
| Allergy & Immunology | Medical Specialty |
| Pediatric Emergency Medicine | Medical Specialty |
| Nephrology | Medical Specialty |
| Ped Aller-Immuno-Pul | Medical Specialty |
| Ped. Neuro Oncology | Medical Specialty |
| Ped Neonatology | Neonatology |
| Newborn | Neonatology |
| Ped Critical Care | Neonatology |
| Intensive Care | Neonatology |
| Pediatric Observation | Neonatology |
| Critical Care Medicine | Neonatology |
| Obstetrics-Observation | Neonatology |
| General Pediatrics | Pediatrics |
| Pediatric Private | Pediatrics |
| Pediatric Semi-Private | Pediatrics |
| Obstetrics | Pediatrics |
| General Pediatrics-DEL | Pediatrics |
| Labor and Delivery | Pediatrics |
| Family Medicine | Pediatrics |
| Pediatric Surgery | Surgical Specialty |
| Neurosurgery | Surgical Specialty |
| Plastic Surgery | Surgical Specialty |
| Ear Nose Throat | Surgical Specialty |
| Urology | Surgical Specialty |
| Cardio-Thoracic Surgery | Surgical Specialty |
| General Surgery | Surgical Specialty |
| Ped Bone Marrow Tran | Surgical Specialty |
| Orthopedics | Surgical Specialty |
| Transplant | Surgical Specialty |
| No Data | Unknown |

**eTable 9:** Group Definitions for Inpatient Services

| **Clinic Service or Specialty** | **Clinic Service Group** | **Clinic Service Subgroup** |
| --- | --- | --- |
| PED. BEHAVIORAL DEVELOPMENT & GEN | Developmental-Behavioral Pediatrics | Developmental-Behavioral Pediatrics |
| Pediatric Genetics | Developmental-Behavioral Pediatrics | Developmental-Behavioral Pediatrics |
| Pediatric Neurodevelopment | Developmental-Behavioral Pediatrics | Developmental-Behavioral Pediatrics |
| PED BEHAV DEV-GEN | Developmental-Behavioral Pediatrics | Developmental-Behavioral Pediatrics |
| Psychiatry | Developmental-Behavioral Pediatrics | Developmental-Behavioral Pediatrics |
| Developmental and Behavioral Pediatrics | Developmental-Behavioral Pediatrics | Developmental-Behavioral Pediatrics |
| CHART RESPONSIBLE MD | CHART RESPONSIBLE MD | None |
| Child Abuse and Neglect | Child Abuse and Neglect | None |
| Lab | Diagnostics | Lab |
| Pre-Admission Testing | Diagnostics | Lab |
| Pathology | Diagnostics | Lab |
| Radiology | Diagnostics | Radiology |
| Pediatric Cardiology | Medical Specialty | Cardiology |
| GENERAL MEDICINE | Medical Specialty | Medicine |
| Dermatology | Medical Specialty | Dermatology |
| Pediatric Neurology | Medical Specialty | Neurology |
| Pediatric Gastroenterology | Medical Specialty | Gastroenterology |
| Pediatric Pulmonology | Medical Specialty | Allergy and Pulmonology |
| Cardiology | Medical Specialty | Cardiology |
| Pediatric Infectious Disease | Medical Specialty | Medicine |
| Pediatric Endocrinology | Medical Specialty | Endocrinology |
| Pediatric Dermatology | Medical Specialty | Dermatology |
| PEDIATRIC METABOLISM | Medical Specialty | Endocrinology |
| Pediatric Nephrology | Medical Specialty | Other |
| PED. ALLERGY / IMMUNOLOGY /PULMON | Medical Specialty | Allergy and Pulmonology |
| Pediatric Allergy and Immunology | Medical Specialty | Allergy and Pulmonology |
| Neurology | Medical Specialty | Neurology |
| Pediatric Allergy | Medical Specialty | Allergy and Pulmonology |
| Pulmonary and Allergy | Medical Specialty | Allergy and Pulmonology |
| Pediatric Sleep | Medical Specialty | Other |
| Gastroenterology | Medical Specialty | Gastroenterology |
| Nephrology | Medical Specialty | Other |
| Pediatric Transplant | Medical Specialty | Other |
| GYN ENDOCRINE | Medical Specialty | Endocrinology |
| General Internal Medicine | Medical Specialty | Medicine |
| Sleep Medicine | Medical Specialty | Other |
| Internal Medicine | Medical Specialty | Medicine |
| Podiatry | Medical Specialty | Other |
| PEDIATRIC HEMATOLOGY / ONCOLOGY | Medical Specialty | Heme/Onc |
| PHO-Pediatric Hematology and Oncology | Medical Specialty | Heme/Onc |
| Oncology | Medical Specialty | Heme/Onc |
| PEDIATRIC NEURO-ONCOLOGY | Medical Specialty | Neurology |
| Hematology | Medical Specialty | Heme/Onc |
| Neonatology | Neonatology | Neonatology |
| Perinatology | Neonatology | Neonatology |
| Pediatric Intensive Care | Neonatology | Neonatology |
| Obstetrics | Neonatology | Neonatology |
| Gynecology | Neonatology | Neonatology |
| Obstetrics and Gynecology | Neonatology | Neonatology |
| OBSTETRICS-OBSERVATION | Neonatology | Neonatology |
| Ophthalmology | Ophthalmology | Ophthalmology |
| Physical and Occupational Therapy | Physical Therapy | Physical Therapy |
| Pediatric Physical Therapy | Physical Therapy | Physical Therapy |
| PEDIATRIC REHABILITATION | Physical Therapy | Physical Therapy |
| Sports Medicine | Physical Therapy | Physical Therapy |
| Pediatrics | Pediatrics and Primary Care | Pediatrics and Primary Care |
| COMMUNITY AND FAMILY MEDICINE | Pediatrics and Primary Care | Pediatrics and Primary Care |
| Primary Care | Pediatrics and Primary Care | Pediatrics and Primary Care |
| Family Medicine | Pediatrics and Primary Care | Pediatrics and Primary Care |
| Healthy Lifestyles | Pediatrics and Primary Care | Pediatrics and Primary Care |
| Speech Pathology | Speech and Hearing | Speech Pathology |
| Pediatric Audiology | Speech and Hearing | Audiology |
| Speech Therapy | Speech and Hearing | Speech Pathology |
| Pediatric Speech Therapy | Speech and Hearing | Speech Pathology |
| Audiology | Speech and Hearing | Audiology |
| PBMT-Pediatric Bone Marrow Transplant | Surgical Specialty | Other |
| Radiation Oncology | Surgical Specialty | Other |
| Urology | Surgical Specialty | Urology |
| Orthopaedics | Surgical Specialty | Orthopaedics |
| General Surgery | Surgical Specialty | General Surgery |
| EAR NOSE THROAT | Surgical Specialty | Ear, Nose, and Throat |
| Pediatric Surgery | Surgical Specialty | General Surgery |
| Pediatric Urology | Surgical Specialty | Urology |
| Pediatric Otolaryngology | Surgical Specialty | Ear, Nose, and Throat |
| Plastic Surgery | Surgical Specialty | Plastic Surgery |
| Neurosurgery | Surgical Specialty | Neurosurgery |
| Pediatric Orthopaedics | Surgical Specialty | Orthopaedics |
| Pediatric Plastic Surgery | Surgical Specialty | Plastic Surgery |
| Pediatric Neurosurgery | Surgical Specialty | Neurosurgery |
| Otolaryngology | Surgical Specialty | Ear, Nose, and Throat |
| Thoracic Surgery | Surgical Specialty | Other |
| Wound Care | Surgical Specialty | Other |
| Pediatric Anesthesia | Surgical Specialty | Other |
| ORTHOPAEDIC TRAUMA SERVICE | Surgical Specialty | Other |
| Pediatric Cardiothoracic Surgery | Surgical Specialty | Other |
| Transplant | Surgical Specialty | Other |
| TRAUMA | Surgical Specialty | Other |
| Missing or invalid | Unknown | None |
| OUTPATIENT | Unknown | None |
| No Match | Unknown | None |
| Inpatient | Unknown | None |
| No Data | Unknown | None |
| PEDIATRIC PRIVATE | Unknown | None |
| Emergency Medicine | Urgent Care | None |
| Urgent Care | Urgent Care | None |

**eTable 10:** Group Definitions for Procedures

| **Clinical Classification Software**  **(CCS) Category** | **Multi CCS Level 1** | **Procedure Category** |
| --- | --- | --- |
| Blood transfusion | Miscellaneous diagnostic and therapeutic procedures | Blood Transfusion |
| Conversion of cardiac rhythm | Miscellaneous diagnostic and therapeutic procedures | Cardiovascular Procedures |
| Other OR procedures on vessels other than head and neck | Operations on the cardiovascular system | Cardiovascular Procedures |
| Other OR heart procedures | Operations on the cardiovascular system | Cardiovascular Procedures |
| Extracorporeal circulation auxiliary to open heart procedures | Operations on the cardiovascular system | Cardiovascular Procedures |
| Diagnostic cardiac catheterization; coronary arteriography | Operations on the cardiovascular system | Cardiovascular Procedures |
| Other non-OR therapeutic cardiovascular procedures | Operations on the cardiovascular system | Cardiovascular Procedures |
| Heart valve procedures | Operations on the cardiovascular system | Cardiovascular Procedures |
| Other vascular bypass and shunt; not heart | Operations on the cardiovascular system | Cardiovascular Procedures |
| Aortic resection; replacement or anastomosis | Operations on the cardiovascular system | Cardiovascular Procedures |
| Other diagnostic cardiovascular procedures | Operations on the cardiovascular system | Cardiovascular Procedures |
| Insertion; revision; replacement; removal of cardiac pacemaker or cardioverter/defibrillator | Operations on the cardiovascular system | Cardiovascular Procedures |
| Other OR procedures on vessels of head and neck | Operations on the cardiovascular system | Cardiovascular Procedures |
| Percutaneous transluminal coronary angioplasty  (PTCA) with or without stent placement | Operations on the cardiovascular system | Cardiovascular Procedures |
| Hemodialysis | Operations on the cardiovascular system | Cardiovascular Procedures |
| Circumcision | Operations on the male genital organs | Circumcision |
| Other OR therapeutic procedures; male genital | Operations on the male genital organs | Circumcision |
| Other non-OR therapeutic procedures; male genital | Operations on the male genital organs | Circumcision |
| Other diagnostic radiology and related techniques | Miscellaneous diagnostic and therapeutic procedures | Diagnostic Imaging |
| Magnetic resonance imaging | Miscellaneous diagnostic and therapeutic procedures | Diagnostic Imaging |
| Routine chest X-ray | Miscellaneous diagnostic and therapeutic procedures | Diagnostic Imaging |
| Cancer chemotherapy | Miscellaneous diagnostic and therapeutic procedures | Diagnostic Imaging |
| Diagnostic ultrasound of abdomen or retroperitoneum | Miscellaneous diagnostic and therapeutic procedures | Diagnostic Imaging |
| CT scan head and neck | Miscellaneous diagnostic and therapeutic procedures | Diagnostic Imaging |
| Arterio- or venogram  (not heart and head/neck) | Miscellaneous diagnostic and therapeutic procedures | Diagnostic Imaging |
| Upper gastrointestinal X-ray | Miscellaneous diagnostic and therapeutic procedures | Diagnostic Imaging |
| Contrast aortogram | Miscellaneous diagnostic and therapeutic procedures | Diagnostic Imaging |
| Other diagnostic ultrasound | Miscellaneous diagnostic and therapeutic procedures | Diagnostic Imaging |
| Diagnostic ultrasound of head and neck | Miscellaneous diagnostic and therapeutic procedures | Diagnostic Imaging |
| Diagnostic ultrasound of gastrointestinal tract | Miscellaneous diagnostic and therapeutic procedures | Diagnostic Imaging |
| Diagnostic ultrasound of urinary tract | Miscellaneous diagnostic and therapeutic procedures | Diagnostic Imaging |
| Other nuclear medicine imaging | Miscellaneous diagnostic and therapeutic procedures | Diagnostic Imaging |
| Cerebral and neck arteriogram | Miscellaneous diagnostic and therapeutic procedures | Diagnostic Imaging |
| Intraoperative cholangiogram | Miscellaneous diagnostic and therapeutic procedures | Diagnostic Imaging |
| Lower gastrointestinal X-ray | Miscellaneous diagnostic and therapeutic procedures | Diagnostic Imaging |
| CT scan abdomen | Miscellaneous diagnostic and therapeutic procedures | Diagnostic Imaging |
| CT scan chest | Miscellaneous diagnostic and therapeutic procedures | Diagnostic Imaging |
| Other CT scan | Miscellaneous diagnostic and therapeutic procedures | Diagnostic Imaging |
| Diagnostic spinal tap | Operations on the nervous system | Diagnostic Spinal Tap |
| Inguinal and femoral hernia repair | Operations on the digestive system | Digestive System Procedures |
| Gastrostomy; temporary and permanent | Operations on the digestive system | Digestive System Procedures |
| Other OR lower GI therapeutic procedures | Operations on the digestive system | Digestive System Procedures |
| Other OR upper GI therapeutic procedures | Operations on the digestive system | Digestive System Procedures |
| Other OR gastrointestinal therapeutic procedures | Operations on the digestive system | Digestive System Procedures |
| Other non-OR upper GI therapeutic procedures | Operations on the digestive system | Digestive System Procedures |
| Ileostomy and other enterostomy | Operations on the digestive system | Digestive System Procedures |
| Small bowel resection | Operations on the digestive system | Digestive System Procedures |
| Upper gastrointestinal endoscopy; biopsy | Operations on the digestive system | Digestive System Procedures |
| Excision; lysis peritoneal adhesions | Operations on the digestive system | Digestive System Procedures |
| Other hernia repair | Operations on the digestive system | Digestive System Procedures |
| Colorectal resection | Operations on the digestive system | Digestive System Procedures |
| Laparoscopy  (GI only) | Operations on the digestive system | Digestive System Procedures |
| Appendectomy | Operations on the digestive system | Digestive System Procedures |
| Proctoscopy and anorectal biopsy | Operations on the digestive system | Digestive System Procedures |
| Colostomy; temporary and permanent | Operations on the digestive system | Digestive System Procedures |
| Other non-OR gastrointestinal therapeutic procedures | Operations on the digestive system | Digestive System Procedures |
| Abdominal paracentesis | Operations on the digestive system | Digestive System Procedures |
| Biopsy of liver | Operations on the digestive system | Digestive System Procedures |
| Esophageal dilatation | Operations on the digestive system | Digestive System Procedures |
| Other non-OR lower GI therapeutic procedures | Operations on the digestive system | Digestive System Procedures |
| Exploratory laparotomy | Operations on the digestive system | Digestive System Procedures |
| Other bowel diagnostic procedures | Operations on the digestive system | Digestive System Procedures |
| Colonoscopy and biopsy | Operations on the digestive system | Digestive System Procedures |
| Excision  (partial) of large intestine  (not endoscopic) | Operations on the digestive system | Digestive System Procedures |
| Cholecystectomy and common duct exploration | Operations on the digestive system | Digestive System Procedures |
| Other gastrointestinal diagnostic procedures | Operations on the digestive system | Digestive System Procedures |
| Diagnostic ultrasound of heart  (echocardiogram) | Miscellaneous diagnostic and therapeutic procedures | Echocardiogram |
| Electrocardiogram | Miscellaneous diagnostic and therapeutic procedures | Electrocardiogram |
| Ophthalmologic and otologic diagnosis and treatment | Miscellaneous diagnostic and therapeutic procedures | ENT and Eye Procedures |
| Other therapeutic procedures on the ear nose and sinus | Operations on the ear | ENT and Eye Procedures |
| Myringotomy | Operations on the ear | ENT and Eye Procedures |
| Diagnostic procedures on ear nose and sinus | Operations on the ear | ENT and Eye Procedures |
| Destruction of lesion of retina and choroid | Operations on the eye | ENT and Eye Procedures |
| Other therapeutic procedures on eyelids; conjunctiva; cornea | Operations on the eye | ENT and Eye Procedures |
| Other intraocular therapeutic procedures | Operations on the eye | ENT and Eye Procedures |
| Lens and cataract procedures | Operations on the eye | ENT and Eye Procedures |
| Other extraocular muscle and orbit therapeutic procedures | Operations on the eye | ENT and Eye Procedures |
| Diagnostic procedures on eye | Operations on the eye | ENT and Eye Procedures |
| Procedures typically performed for glaucoma | Operations on the eye | ENT and Eye Procedures |
| Repair of retina | Operations on the eye | ENT and Eye Procedures |
| Other OR procedures on mouth and throat | Operations on the nose; mouth; and pharynx | ENT and Eye Procedures |
| Diagnostic procedures on mouth and throat | Operations on the nose; mouth; and pharynx | ENT and Eye Procedures |
| Other non-OR procedures on mouth and throat | Operations on the nose; mouth; and pharynx | ENT and Eye Procedures |
| Plastic procedures on nose | Operations on the nose; mouth; and pharynx | ENT and Eye Procedures |
| Tonsillectomy and/or adenoidectomy | Operations on the nose; mouth; and pharynx | ENT and Eye Procedures |
| Dental procedures | Operations on the nose; mouth; and pharynx | ENT and Eye Procedures |
| Enteral and parenteral nutrition | Miscellaneous diagnostic and therapeutic procedures | Enteral and Parenteral Nutrition |
| Nasogastric tube | Miscellaneous diagnostic and therapeutic procedures | Enteral and Parenteral Nutrition |
| Respiratory intubation and mechanical ventilation | Miscellaneous diagnostic and therapeutic procedures | Intubation and Ventilation |
| Traction; splints; and other wound care | Miscellaneous diagnostic and therapeutic procedures | Musculoskeletal System Procedures |
| Other non-OR therapeutic procedures on musculoskeletal system | Operations on the musculoskeletal system | Musculoskeletal System Procedures |
| Other therapeutic procedures on muscles and tendons | Operations on the musculoskeletal system | Musculoskeletal System Procedures |
| Fracture treatment including reposition with or without fixation; radius or ulna fracture or dislocation | Operations on the musculoskeletal system | Musculoskeletal System Procedures |
| Other OR therapeutic procedures on musculoskeletal system | Operations on the musculoskeletal system | Musculoskeletal System Procedures |
| Partial excision bone | Operations on the musculoskeletal system | Musculoskeletal System Procedures |
| Fracture treatment including reposition with or without fixation; lower extremity fracture or dislocation  (other than hip or femur) | Operations on the musculoskeletal system | Musculoskeletal System Procedures |
| Other diagnostic procedures on musculoskeletal system | Operations on the musculoskeletal system | Musculoskeletal System Procedures |
| Division or release of joint capsule; ligament or cartilage | Operations on the musculoskeletal system | Musculoskeletal System Procedures |
| Other OR therapeutic procedures on joints | Operations on the musculoskeletal system | Musculoskeletal System Procedures |
| Amputation of lower extremity | Operations on the musculoskeletal system | Musculoskeletal System Procedures |
| Fracture treatment including reposition with or without fixation; hip or femur fracture or dislocation | Operations on the musculoskeletal system | Musculoskeletal System Procedures |
| Fracture treatment including reposition with or without fixation; facial fracture or dislocation | Operations on the musculoskeletal system | Musculoskeletal System Procedures |
| Other OR therapeutic procedures on bone | Operations on the musculoskeletal system | Musculoskeletal System Procedures |
| Fracture treatment including reposition with or without fixation of other fracture or or dislocation | Operations on the musculoskeletal system | Musculoskeletal System Procedures |
| Arthroplasty other than hip or knee | Operations on the musculoskeletal system | Musculoskeletal System Procedures |
| Other OR therapeutic nervous system procedures | Operations on the nervous system | Nervous System Procedures |
| Insertion; replacement; or removal of extracranial ventricular shunt | Operations on the nervous system | Nervous System Procedures |
| Other non-OR or closed therapeutic nervous system procedures | Operations on the nervous system | Nervous System Procedures |
| Incision and excision of CNS | Operations on the nervous system | Nervous System Procedures |
| Decompression peripheral nerve | Operations on the nervous system | Nervous System Procedures |
| Excision destruction or resection of intervertebral disc | Operations on the nervous system | Nervous System Procedures |
| Other diagnostic nervous system procedures | Operations on the nervous system | Nervous System Procedures |
| Insertion of catheter or spinal stimulator and injection into spinal canal | Operations on the nervous system | Nervous System Procedures |
| Other diagnostic procedures | Miscellaneous diagnostic and therapeutic procedures | Other Diagnostic Procedures |
| Electroencephalogram  (EEG) | Miscellaneous diagnostic and therapeutic procedures | Other Diagnostic Procedures |
| Swan-Ganz catheterization for monitoring | Miscellaneous diagnostic and therapeutic procedures | Other Diagnostic Procedures |
| Arterial blood gases | Miscellaneous diagnostic and therapeutic procedures | Other Diagnostic Procedures |
| Diagnostic physical therapy | Miscellaneous diagnostic and therapeutic procedures | Other Diagnostic Procedures |
| Therapeutic endocrine procedures | Operations on the endocrine system | Other Diagnostic Procedures |
| Other diagnostic procedures; female organs | Operations on the female genital organs | Other Diagnostic Procedures |
| Bone marrow biopsy | Operations on the hemic and lymphatic system | Other Diagnostic Procedures |
| Other procedures; hemic and lymphatic systems | Operations on the hemic and lymphatic system | Other Diagnostic Procedures |
| Other therapeutic procedures | Miscellaneous diagnostic and therapeutic procedures | Other Therapeutic Procedures |
| Nonoperative removal of foreign body | Miscellaneous diagnostic and therapeutic procedures | Other Therapeutic Procedures |
| Other physical therapy and rehabilitation | Miscellaneous diagnostic and therapeutic procedures | Other Therapeutic Procedures |
| Organ transplantation  (other than bone marrow corneal or kidney) | Miscellaneous diagnostic and therapeutic procedures | Other Therapeutic Procedures |
| Physical therapy exercises; manipulation; and other procedures | Miscellaneous diagnostic and therapeutic procedures | Other Therapeutic Procedures |
| Radiation therapy | Miscellaneous diagnostic and therapeutic procedures | Other Therapeutic Procedures |
| Other OR therapeutic procedures; female organs | Operations on the female genital organs | Other Therapeutic Procedures |
| Bone marrow transplant | Operations on the hemic and lymphatic system | Other Therapeutic Procedures |
| Other respiratory therapy | Miscellaneous diagnostic and therapeutic procedures | Respiratory System Procedures |
| Tracheoscopy and laryngoscopy with biopsy | Operations on the respiratory system | Respiratory System Procedures |
| Diagnostic bronchoscopy and biopsy of bronchus | Operations on the respiratory system | Respiratory System Procedures |
| Other OR Rx procedures on respiratory system and mediastinum | Operations on the respiratory system | Respiratory System Procedures |
| Incision of pleura; thoracentesis; chest drainage | Operations on the respiratory system | Respiratory System Procedures |
| Other non-OR therapeutic procedures on respiratory system and mediastinum | Operations on the respiratory system | Respiratory System Procedures |
| Tracheostomy; temporary and permanent | Operations on the respiratory system | Respiratory System Procedures |
| Other diagnostic procedures on the respiratory system and mediastinum | Operations on the respiratory system | Respiratory System Procedures |
| Lobectomy or pneumonectomy | Operations on the respiratory system | Respiratory System Procedures |
| Excision of skin | Operations on the integumentary system | Skin Procedures |
| Other non-OR therapeutic procedures on skin subcutaneous tissue fascia and breast | Operations on the integumentary system | Skin Procedures |
| Incision and drainage; skin subcutaneous tissue and fascia | Operations on the integumentary system | Skin Procedures |
| Other diagnostic procedures on skin subcutaneous tissue fascia and breast | Operations on the integumentary system | Skin Procedures |
| Repair of skin subcutaneous tissue and fascia | Operations on the integumentary system | Skin Procedures |
| Skin graft | Operations on the integumentary system | Skin Procedures |
| Other OR therapeutic procedures on skin subcutaneous tissue fascia and breast | Operations on the integumentary system | Skin Procedures |
| Nonoperative urinary system measurements and monitoring | Miscellaneous diagnostic and therapeutic procedures | Urinary System Procedures |
| Other diagnostic procedures of urinary tract | Operations on the urinary system | Urinary System Procedures |
| Transurethral excision; drainage; or removal urinary obstruction | Operations on the urinary system | Urinary System Procedures |
| Procedures on the urethra | Operations on the urinary system | Urinary System Procedures |
| Other OR therapeutic procedures of urinary tract | Operations on the urinary system | Urinary System Procedures |
| Endoscopy and endoscopic biopsy of the urinary tract | Operations on the urinary system | Urinary System Procedures |
| Indwelling catheter | Operations on the urinary system | Urinary System Procedures |
| Nephrectomy; partial or complete | Operations on the urinary system | Urinary System Procedures |
| Nephrotomy and nephrostomy | Operations on the urinary system | Urinary System Procedures |
| Other non-OR therapeutic procedures of urinary tract | Operations on the urinary system | Urinary System Procedures |
| Prophylactic vaccinations and inoculations | Miscellaneous diagnostic and therapeutic procedures | Vaccinations and Inoculations |
| Other vascular catheterization; not heart | Operations on the cardiovascular system | Vascular Catheterization  (not heart) |
